# Genome-wide identification, molecular evolution and gene expression of *P450* gene family in *Cyrtotrachelus buqueti*

**DOI:** 10.1186/s12864-024-10372-5

**Published:** 2024-05-08

**Authors:** Chun Fu, Ding Yang, Wen Cong Long, XiMeng Xiao, HanYu Wang, Na Jiang, YaoJun Yang

**Affiliations:** 1 Key Laboratory of Sichuan Province for Bamboo Pests Control and Resource Development, Leshan Normal University, No. 778 Binhe Road, Shizhong District, Leshan, 614000 Sichuan China; 2 College of Life Science, Leshan Normal University, No. 778 Binhe Road, Shizhong District, Leshan, 614000 Sichuan China; 3 College of Tourism and Geographical Science, Leshan Normal University, No. 778 Binhe Road, Shizhong District, Leshan, 614000 Sichuan China

**Keywords:** *Cyrtotrachelus buqueti*, *P450* gene family, Molecular evolution, Protein interaction

## Abstract

**Background:**

Insect Cytochrome *P450* monooxygenase (*CYPs* or *P450*s) plays an important role in detoxifying insecticides, causing insect populations to develop resistance. However, the molecular functions of *P450* gene family in *Cyrtotrachelus buqueti* genome are still lacking.

**Results:**

In this study, 71 *CbuP450* genes have been identified. The amino acids length of CbuP450 proteins was between 183 aa ~ 1041 aa. They are proteins with transmembrane domains. The main component of their secondary structure is α-helix and random coils. Phylogenetic analysis showed that *C. buqueti* and *Rhynchophorus ferrugineus* were the most closely related. This gene family has 29 high-frequency codons, which tend to use A/T bases and A/T ending codons. Gene expression analysis showed that *CbuP450_23* in the female adult may play an important role on high temperature resistance, and Cbu*P450*_17 in the larval may play an important role on low temperature tolerance. *CbuP450_10, CbuP450_17, CbuP450_23, CbuP450_10, CbuP450_16, CbuP450_20, CbuP450_23* and *CbuP450_ 29* may be related to the regulation of bamboo fiber degradation genes in *C. buqueti*. Protein interaction analysis indicates that most CbuP450 proteins are mainly divided into three aspects: encoding the biosynthesis of ecdysteroids, participating in the decomposition of synthetic insecticides, metabolizing insect hormones, and participating in the detoxification of compounds.

**Conclusions:**

We systematically analyzed the gene and protein characteristics, gene expression, and protein interactions of *CbuP450* gene family, revealing the key genes involved in the stress response of *CbuP450* gene family in the resistance of *C. buqueti* to high or low temperature stress, and identified the key CbuP450 proteins involved in important life activity metabolism. These results provided a reference for further research on the function of *P450* gene family in *C. buqueti*.

**Supplementary Information:**

The online version contains supplementary material available at 10.1186/s12864-024-10372-5.

## Background

Cytochrome *P450* is a class of metabolic enzymes widely present in aerobic organisms that catalyze various NADPH and ATP-dependent oxidation, dealkylation, and dehydrogenation. Six cytochrome P450 enzymes are involved in human steroidogenesis, converting cholesterol to sex steroids, mineralocorticoids, and glucocorticoids. Human cytochrome P450 enzyme 1A2 (CYP1A2) metabolises many clinical drugs, such as phenacetin, caffeine, clozapine, tacrine, propranolol, and mexiletine. CYP1A2 also metabolises certain precarcinogens such as aflatoxins, mycotoxins, nitrosamines, and endogenous substances such as steroids [1]. It regulates the metabolism of a variety of endogenous substances and exogenous compounds [2], and is important in the metabolism of xenobial organisms and in the production of compounds such as cyanoglucosides, and many insects are used for defense [3]. However, the molecular basis for the transcriptional regulation of Cytochrome *P450* remains largely unknown [4]. Studies have shown that it is difficult to utilize *P450* effectively now because they must be paired with cytochrome *P450* reductase (CPRs) to facilitate electron transfer from reduced nicotinamide adenine dinucleotide phosphoric acid (NADPH) [5]. Insects are the most prosperous biological population on the earth, the role of *P450* involves insect growth, development, feeding and other processes, it is also involved in the synthesis and metabolism of many endogenous compounds in insects, and metabolic detoxification is one of the important functions of the enzyme system. There are two possible mechanisms for the enhancement of the metabolic detoxification effect of resistant insect *P450*, one is the increase in the catalytic activity of *P450*, and the other is the increase in the amount of *P450* enzymes. The root cause of the increase in catalytic activity lies in changes in the structure of *P450*, which may involve amino acid replacement, selective splicing, or chimera formation, while an increase in *P450* enzyme volume involves an increase in the number of copies of *P450* gene and an upregulation of the expression of *P450* genes [6]. *P450* exhibits tissue-specific or different modes of expression that support their primary role in detoxification and/or digestion in specific tissues [7]. It is known that the fat body is the main detoxification and metabolic site of insects [8] in terms of exogenous action, the *P450* system can convert toxic lipophilic substances into more hydrophilic metabolites, which are easily removed by the organism’s water-based excretion system. This detoxification ability is evident in organisms, especially herbivores, to address the need for detoxification of plant-based defensive toxins such as tannins, phenols, quinones, alkaloids, steroids, lecithin, and lignin [9]. Previously, studies have shown that CYP6B7 from Lepidoptera noctuidae (HaCYP6B7) is expressed in *Pichia pastoris* GS115 strain and has been shown to detoxify bifenthrin, fenvalle ester, and chlorpyrifos [10]. Among insects, the *P450* has a variety of functions. For example, the isolated cytochrome *P450* gene *AccCYP336A1* may play a very important role in antioxidant defense against reactive oxygen species (ROS) damage [11]. Sequencing in all regions of the Tree of Life has yielded > 300,000 Cytochrome *P450 (CYP)* sequences that have been mined and collected. The nomenclature has been assigned to > 41,000 CYP sequences, most of the rest have been classified into clans, families, and subfamilies by BLAST search for designation, and the *P450* sequence space is being systematically explored and populated [12]. More than 200 insect cytochrome *P450* gene fragments have been identified, belonging to *CYP4, CYP6, CYP9, CYP12, CYP15, CYP18, CYP28, CYP48, CYP305* and other gene families, which are also related to larval hormones and molting steroid biosynthetic pathways, which are in the central stage of insect growth, development and reproduction [9]. It is known that *P450* genes are involved in the production of chemical signals in cockroaches, Their expression is associated with one of the main fertility regulators, the young hormones. This makes the *P450* gene, in the face of natural selection, which makes the *P450* gene an evolutionary substrate for the production of both suitable and usable in the face of natural selection [13]. The insect monooxygenase system is involved in the oxidative metabolism of endogenous and exogenous substrates, and is important in the growth and development of insects, a variety of edible plants adapted to insectivorous plants, and insecticides [14]. Across the insect genome, the size of Cytochrome *P450* monooxygenase *(CYP)* gene superfamily varies widely. Changes in *CYPome* size are attributed to mutually adaptive radiation in insect detoxification genes in response to co-evolutionary radiation from plant biosynthetic genes driven by herbivores and their chemically defended host plants [15].

At present, we know more than 1 million species of insects, accounting for more than 50% of all biological species, of which there are more than 330,000 species of Coleoptera, which is the first order in the insect order. The main feature is their special forewing, which has become a hard elytra, covering the flying hindwings. At present, there are not many studies on the *P450* gene of Coleoptera, among which Trichophylla, as an important model insect, is the first Coleoptera to complete genome sequencing. Some of these species are important pests in agriculture, forestry, fruit trees and horticulture, so the use of pesticides is an important means of reducing plant damage. Changes in detoxification enzyme activity and associated gene expression in insects have a strong impact on detoxification metabolism and insecticide resistance [16] An important mechanism of insect resistance to insecticides is increased by cytochrome *P450* microsomal monooxygenase-mediated detoxification [17]. Insect Cytochrome *P450* monooxygenase (*CYPs* or *P450*s) plays an important role in detoxifying insecticides, causing insect populations to develop resistance [18], and the frequent and sustained use of insecticides inevitably leads to insect resistance, where the excessive expression of cytochrome *P450* involved in the detoxification of insecticides is an important reason for insects to develop resistance to different types of insecticides. It is also one of the largest enzyme superfamilyses found in nature, catalyzing the conversion of lipophilic compounds (endogenous or xenomorphs) into more hydrophilic derivatives [19]. Therefore, studies have shown that pipeributyl alcohol (*PBO*) is an insecticide synergist that inhibits the activity of Cytochrome *P450* enzyme, which is currently used in some pesticide formulations and is also recommended as a pretreatment agent for certain pesticide applications, which can more effectively control pests [20]. At the same time, different *P450*s exhibit different responses to odorants, suggesting that specific modulations of *P450* expression by odorants may modulate the sensitivity of olfactory responses to various chemicals [21]. *P450* enzyme can also highly selectively catalyze the oxidation of inert C-H bonds in complex organic compounds under mild conditions, which has the advantages of chemical catalysts, so it has a broad application space in the field of microbial pharmaceuticals [22]. The research of *P450* has extensive practical significance in theoretically exploring the physiological metabolism of organisms, the evolution of selection and the relationship between organisms and the environment, or in the applications of environmental protection, agroecology, biological control, crop genetic engineering and medicine and health, so it should receive greater attention and attention.

Although there are many studies on *P450* gene family, sequencing a large number of insect genomes has found dynamic changes in the number and characteristics of *P450* genes in different insect cytochromes. In an evolutionary sense, rapid birth and death of many *P450* genes has been observed, and only a few homologies exist between *P450* genes and the sequenced insect genomes. Chun Fu et al. have sequenced the whole genome of *C. buqueti* and completed the exploration of specific gene functions such as key genes for bamboo fiber degradation and detoxification enzyme genes [23]. However, there have been no reports on the molecular functions of *P450* gene family in *C. buqueti* genome. In this study, *P450* gene family of *C. buqueti* was identified and the genetic and protein characteristics of this gene family have been systematically analyzed, which including physicochemical characteristics analysis, signal peptide and subcellular localization analysis, hydrophobicity prediction, transmembrane domain analysis, phosphorylation site and secondary structure analysis, molecular evolution analysis, and chromosomal localization analysis. These results have laid a certain theoretical foundation for further study of the function and regulation mechanism of *P450* genes in *C. buqueti*.

## Results

### Identification and physicochemical properties analysis of CbuP450 proteins

The results of gene family identification and analysis indicate that there are 71 *P450* genes(*CbuP450*) in *C. buqueti* genome. The physicochemical properties of CbuP450 proteins were analyzed by using the Pratoparam online program, including protein length, molecular weight, isoelectricity index, instability index, total number of positive (negative) electrical residues, total mean hydrophilic coefficient and other indicators. The physicochemical properties of CbuP450 proteins show that there are great differences in gene length and isoelectric points in different species. As shown in Supplementary Table 1, the amino acid length of CbuP450 proteins was between 183 aa and 1041 aa, and the average length is 515 aa. The molecular weight is between 21.41 and 121 kD, the isoelectric point(pI) is between 5.93 and 9.41, and 91.5% is greater than 7. The instability factors showed that CbuP450_2, CbuP450_3, CbuP450_4, CbuP450_8, CbuP450_9, CbuP450_10, CbuP450_12, CbuP450_13, CbuP450_15, CbuP450_16, CbuP450_17, CbuP450_18, CbuP450_20, CbuP450_25, CbuP450_26, CbuP450_27, CbuP450_28, CbuP450_29, CbuP450_30, CbuP450_31, CbuP450_33, CbuP450_34, CbuP450_35, CbuP450_36, CbuP450_40, CbuP450_41, CbuP450_42, CbuP450_44, CbuP450_46, CbuP450_48, CbuP450_49, CbuP450_50, CbuP450_51, CbuP450_53, CbuP450_55, CbuP450_57, CbuP450_59, CbuP450_62, CbuP450_67, CbuP450_69, CbuP450_70, CbuP450_71 are stable proteins, and the rest are unstable proteins. The total number of negatively charged residues with three CbuP450 members is equal to the total number of positively charged residues, CbuP450_56, CbuP450_59, CbuP450_60 showing electrical neutrality. The total number of residues with negative charges with 5 CbuP450 proteins is greater than the total number of residues with positive charges, showing the negative charge. The total number of negatively charged residues with 63 CbuP450 proteins is less than the total number of positively charged residues, showing positive charges (Supplementary Table 1).

### Signal peptide, subcellular localization and hydrophobicity prediction of CbuP450 proteins

7 CbuP450 proteins including CbuP450_7, CbuP450_11, CbuP450_12, CbuP450_21, CbuP450_36, CbuP450_37 and CbuP450_57 have signal peptides and have been presumed to be secreted proteins, and the rest are non-secretory proteins. Subcellular localization analysis showed that CbuP450_5 was present in the plasma membrane, atomic nucleus, mitochondria; CbuP450_7 was present in the plasma membrane and cytoplasm; Cbu*P450*_11 was present in the plasma membrane, mitochondria, and nuclear membrane; CbuP450_12, CbuP450_46, CbuP450_47 were present in the plasma membrane and nuclear membrane; CbuP450_13 was present in the plasma membrane, cytoplasm, and nuclear membrane; Cbu*P450*_16 was present in the nucleus, cytoplasm, and mitochondria, CbuP450_17, CbuP450_23, CbuP450_44, CbuP450_45, CbuP450_50, CbuP450_61, CbuP450_65 exist only in the cytoplasm; CbuP450_55, CbuP450_62, CbuP450_63, CbuP450_66 only in mitochondria; CbuP450_59 only in the nucleus; CbuP450_39 and CbuP450_67 were found in the plasma membrane and mitochondria; CbuP450_60 and CbuP450_68 were found in the cytoplasm and nuclear membrane; CbuP450_64 were found in the cytoplasm and mitochondria, and the rest were only present in the plasma membrane (Supplementary Table 1).

The hydrophobicity prediction analysis showed that overall distribution of CbuP450 proteins were overwhelmingly negative, indicating that they were hydrophilic proteins, except for CbuP450_32 and CbuP450_71. The maximum hydrophobicity value range was between 1.956 and 4.189, the largest was CbuP450_21, the smallest was CbuP450_68; the minimum hydrophilic value range was between − 2.322 and − 3.478, the smallest is CbuP450_58, and the largest was CbuP450_58. Among them, the average hydrophilic coefficient of CbuP450_55 was the smallest, indicating that its hydrophilicity was the strongest, and its maximum hydrophobicity position was 271, with a value of 2.033; the minimum hydrophobicity position was 381, and the value was − 2.889 (Supplementary Table 1).

### Transmembrane domain structure and phosphorylation site analysis of CbuP450 proteins

Transmembrane domain structure analysis showed that there were 39 CbuP450 proteins contain transmembrane domain structure. CbuP450_4 had 3 transmembrane domains, CbuP450_1, CbuP450_2, CbuP450_3, CbuP450_10, CbuP450_19, CbuP450_25, CbuP450_28, CbuP450_41, CbuP450_42, CbuP450_43 and CbuP450_49 had 2 transmembrane domains, CbuP450_6, CbuP450_12, CbuP450_13, CbuP450_14, CbuP450_15, CbuP450_16, CbuP450_18, CbuP450_20, CbuP450_21, CbuP450_22, CbuP450_23, CbuP450_24, CbuP450_26, CbuP450_29, CbuP450_30, CbuP450_32, CbuP450_36, CbuP450_37, CbuP450_46, CbuP450_51, CbuP450_48, CbuP450_57, CbuP450_61, CbuP450_67, CbuP450_69, CbuP450_70 and CbuP450_71 had 1 transmembrane domain (Supplementary Fig. 1).

The phosphorylation site analysis found that 71 CbuP450 proteins had the largest number of serine phosphorylation sites, followed by threonine and tyrosine. The amino acid most likely to be the potential phosphorylation site is serine, which has reached a minimum of 0.898, far exceeding the standard value of 0.5. Among them, the CbuP450_4 has the largest number of serine, threonine and tyrosine phosphorylation sites, with 93, 49 and 25 respectively. These results suggest that the biological function of CbuP450 proteins requires phosphorylation at the serine site to achieve its biological function. This is consistent with the central dogma of molecular biology, which states that proteins require post-translational modifications to have biological activity (Supplementary Table 1).

### Secondary and tertiary structure analysis of CbuP450 proteins

Secondary structure analysis showed that α-helix accounted for the largest proportion in CbuP450 proteins, reaching 58.71% at the largest. The second is the random coil and extension chain, and the β-turn accounts for the smallest proportion in CbuP450 proteins, with a minimum of 1.09%. The secondary structure of CbuP450 proteins was relatively neat, except for the CbuP450_3 of β-turn > α-helix > random coil > extended chain, the rest were α-helix > random coil > extended chain > β-turn (Supplementary Table 4, Supplementary Fig. 2).

Tertiary structural analysis of CbuP450 proteins showed that its protein members contained α-helix, extended chain, β-turn, random coil and other structures. After analysis and comparison, according to the similarity of tertiary structure, tertiary structure of these 71 CbuP450 members was divided into 14 groups, of which 8 had only one member, namely CbuP450_45, CbuP450_50, CbuP450_53, CbuP450_55, CbuP450_59, CbuP450_69, CbuP450_71 indicating that no other members were also similar to their tertiary structure. The first and second groups had the largest number of them, with 20 each. The first group includes: CbuP450_6, CbuP450_9, CbuP450_11, CbuP450_12, CbuP450_15, CbuP450_23, CbuP450_31, CbuP450_39, CbuP450_46, CbuP450_47, CbuP450_52, CbuP450_57. The second group consisted of CbuP450_2, CbuP450_5, CbuP450_10, CbuP450_13, CbuP450_14, CbuP450_17, CbuP450_28, CbuP450_32, CbuP450_35, CbuP450_37, CbuP450_40, CbuP450_43, CbuP450_48, CbuP450_49, CbuP450_51, CbuP450_54, CbuP450_58, CbuP450_70. The third group consists of 10 CbuP450 proteins, namely CbuP450_26, CbuP450_29, CbuP450_33, CbuP450_60, CbuP450_61, CbuP450_63, CbuP450_67. The fourth group contains: CbuP450_4, CbuP450_25, CbuP450_27, CbuP450_34, and the sixth ancestor contains: CbuP450_3, CbuP450_36, CbuP450_44, CbuP450_56, and the two groups contain the same number. The fifth group, which had only one more member than the previous two, contains: CbuP450_1, CbuP450_24, CbuP450_30, CbuP450_38, CbuP450_62 (Supplementary Fig. 3).

### Conservative motif analysis of CbuP450 proteins

Conservative motif analysis showed that most of 71 CbuP450 proteins had Motif1 to Motif10, except for CbuP450_1, CbuP450_5, CbuP450_38, CbuP450_50, CbuP450_60 only had Motif1, Motif2, Motif3, Motif8; CbuP450_6, CbuP450_9, CbuP450_15, CbuP450_16, CbuP450_19, CbuP450_23, CbuP450_39, CbuP450_46, CbuP450_53 without Motif7, Motif9, Motif10; CbuP450_12 without Motif8 ~ Motif10; CbuP450_24, CbuP450_26, CbuP450_30, CbuP450_32 didn’t have Motif10; CbuP450_11, CbuP450_31, CbuP450_47, CbuP450_49, CbuP450_52 didn’t have Motif6, Motif7, Motif9, Motif10; CbuP450_40 didn’t have Motif7 and Motif9; CbuP450_45 don’t have Motif5, Motif7, Motif9, Motif10; CbuP450_48 didn’t have Motif8, Motif10; CbuP450_54, CbuP450_55, CbuP450_58, CbuP450_59, CbuP450_63 ~ CbuP450_66 don’t have Motif5 ~ Motif7, Motif9 ~ Motif10; CbuP450_57 only had Motif1, Motif2, Motif5, Motif8, CbuP450_67 has more Motif5 than it; CbuP450_62 only had Motif1 ~ Motif4, CbuP450_67 has more Motif5 than it; CbuP450_68 only Motif1, Motif2, Motif4, Motif8; CbuP450_69 didn’t have Motif1, Motif2, Motif4; CbuP450_70 only had Motif2, Motif4, Motif8; CbuP450_71 only have Motif5 ~ Motif9 (Supplementary Fig. 4).

### Gene structure and chromosome localization analysis of *CbuP450* gene family

Gene structure analysis showed that 16 *CbuP450* genes had 7 exons and 6 introns, including *CbuP450_11, CbuP450_13, CbuP450_14, CbuP450_17, CbuP450_24, CbuP450_26, CbuP450_29, CbuP450_30, CbuP450_32, CbuP450_34, CbuP450_35, CbuP450_48, CbuP450_50, CbuP450_51, CbuP450_56, CbuP450_71;* 12 *CbuP450* genes had 8 exons and 7 introns, including *CbuP450_6, CbuP450_8, CbuP450_9, CbuP450_12, CbuP450_16, CbuP450_37, CbuP450_43, CbuP450_44, CbuP450_46, CbuP450_47, CbuP450_58, CbuP450_66;* 8 *CbuP450* genes had 9 exons and 8 introns, including *CbuP450_15, CbuP450_20, CbuP450_21, CbuP450_31, CbuP450_33, CbuP450_38, CbuP450_39, CbuP450_55*; There were 8 *CbuP450* genes with 3 exons and 2 introns, including *CbuP450_10, CbuP450_25, CbuP450_27, CbuP450_28, CbuP450_36, CbuP450_40, CbuP450_57, CbuP450_70*; 6 *CbuP450* genes had 12 exons and 11 introns, including *CbuP450_19, CbuP450_22, CbuP450_23, CbuP450_49, CbuP450_52, CbuP450_64;* 5 *CbuP450* genes had 6 exons and 5 introns, including *CbuP450_2, CbuP450_3, CbuP450_61, CbuP450_63, CbuP450_67*; There were 4 *CbuP450* genes with 10 exons and 9 introns, including *CbuP450_45, CbuP450_54, CbuP450_62, CbuP450_65*; 3 *CbuP450* genes with 4 exons and 3 introns, including *CbuP450_41, CbuP450_59, CbuP450_68*; and 3 *CbuP450* genes with 5 exons and 4 introns, including *CbuP450_5, CbuP450_42, CbuP450_ 69*; 2 *CbuP450* genes had 14 exons and 13 introns, including *CbuP450_4 and CbuP450_6*; 2 *CbuP450* genes have 11 exons and 10 introns, including *CbuP450_7 and CbuP450_53*; and 1 *CbuP450* gene has 18 exons and 17 introns, the largest number of exons and introns in the family, and this member is *CbuP450_1* (Supplementary Fig. 5).

**Fig. 1:**
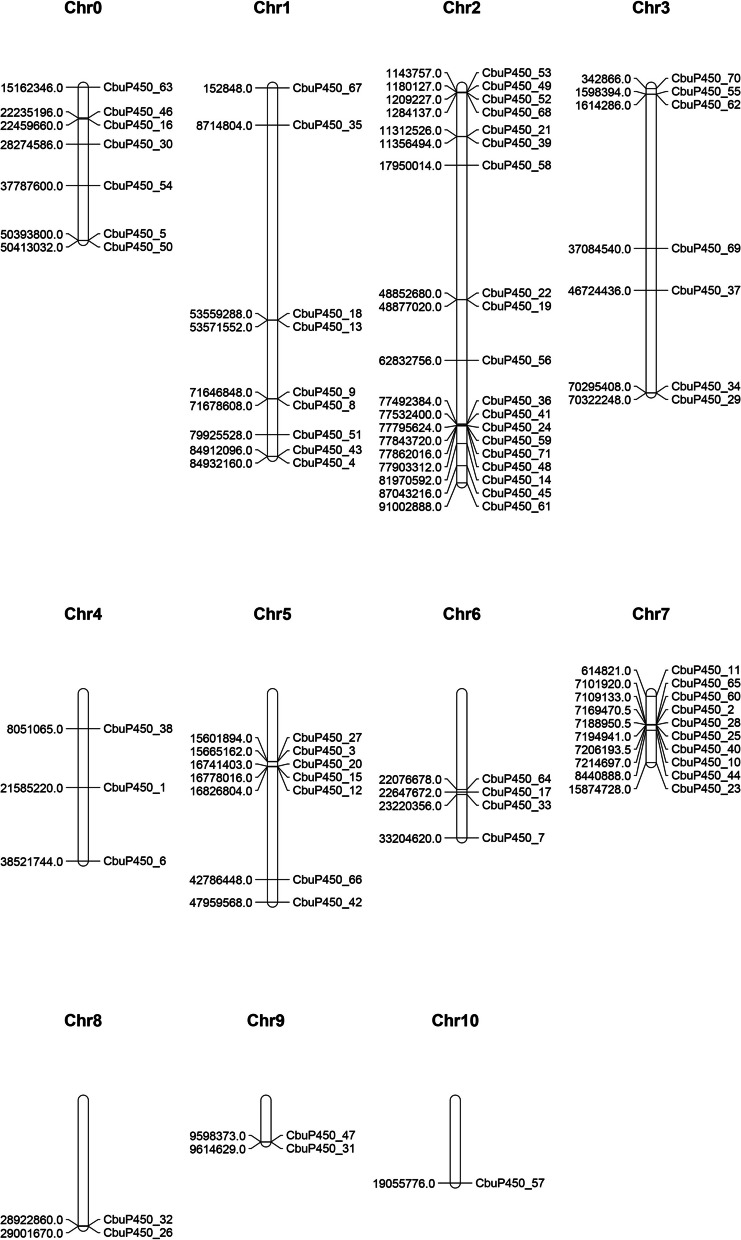
Chromosome mapping analysis of *P450* gene family in *C. buqueti*

Chromosome localization analysis showed that 71 *CbuP450* genes were unevenly distributed on 11 chromosomes, of which Chr2 had the largest number of genes, with 19 *CbuP450* genes, namely *CbuP450_53, CbuP450_49, CbuP450_52, CbuP450_68, CbuP450_21, CbuP450_39, CbuP450_58, CbuP450_22, CbuP450_19, CbuP450_56*, and *CbuP450_36, CbuP450_41, CbuP450_24, CbuP450_59, CbuP450_71, CbuP450_48, CbuP450_14, CbuP450_45, CbuP450_61; Chr0* had a total of 7 *CbuP450* genes, namely *CbuP450_63, CbuP450_46, CbuP450_16, CbuP450_30, CbuP450_54, CbuP450_5, CbuP450_50*; Chr1 had a total of 9 *CbuP450* genes including *CbuP450_67, CbuP450_35, CbuP450_18, CbuP450_13, CbuP450_9, CbuP450_8, CbuP450_51, CbuP450_43, CbuP450_4;* Chr3 had a total of 7 *CbuP450* genes including *CbuP450_70, CbuP450_55, CbuP450_62, CbuP450_69, CbuP450_37, CbuP450_34, CbuP450_29* ; Chr4 had 3 *CbuP450* genes including *CbuP450_38, CbuP450_1 and CbuP450_6*; Chr5 had 7 *CbuP450* genes including *CbuP450_27, CbuP450_3, CbuP450_20, CbuP450_15, CbuP450_12, CbuP450_66 and CbuP450_42*; Chr6 had a total of 4 *CbuP450* genes including *CbuP450_ 64. CbuP450_17, CbuP450_33, CbuP450_7* ;Chr7 had 10 *CbuP450* genes including *CbuP450_11, CbuP450_65, CbuP450_60, CbuP450_2, CbuP450_28, CbuP450_25, CbuP450_40, CbuP450_10, CbuP450_44* and *CbuP450_23*; Chr8 had two *CbuP450* genes including *CbuP450_32 and CbuP450_26*; and Chr9 also had 2 *CbuP450* genes including *CbuP450_ 47 and CbuP450_31*; Chr10 had only one *CbuP450* gene, was CbuP450_57, and the least of these 11 chromosomes (Fig. 1).

### Molecular evolutionary analysis of *CbuP450* gene family

**Fig. 2:**
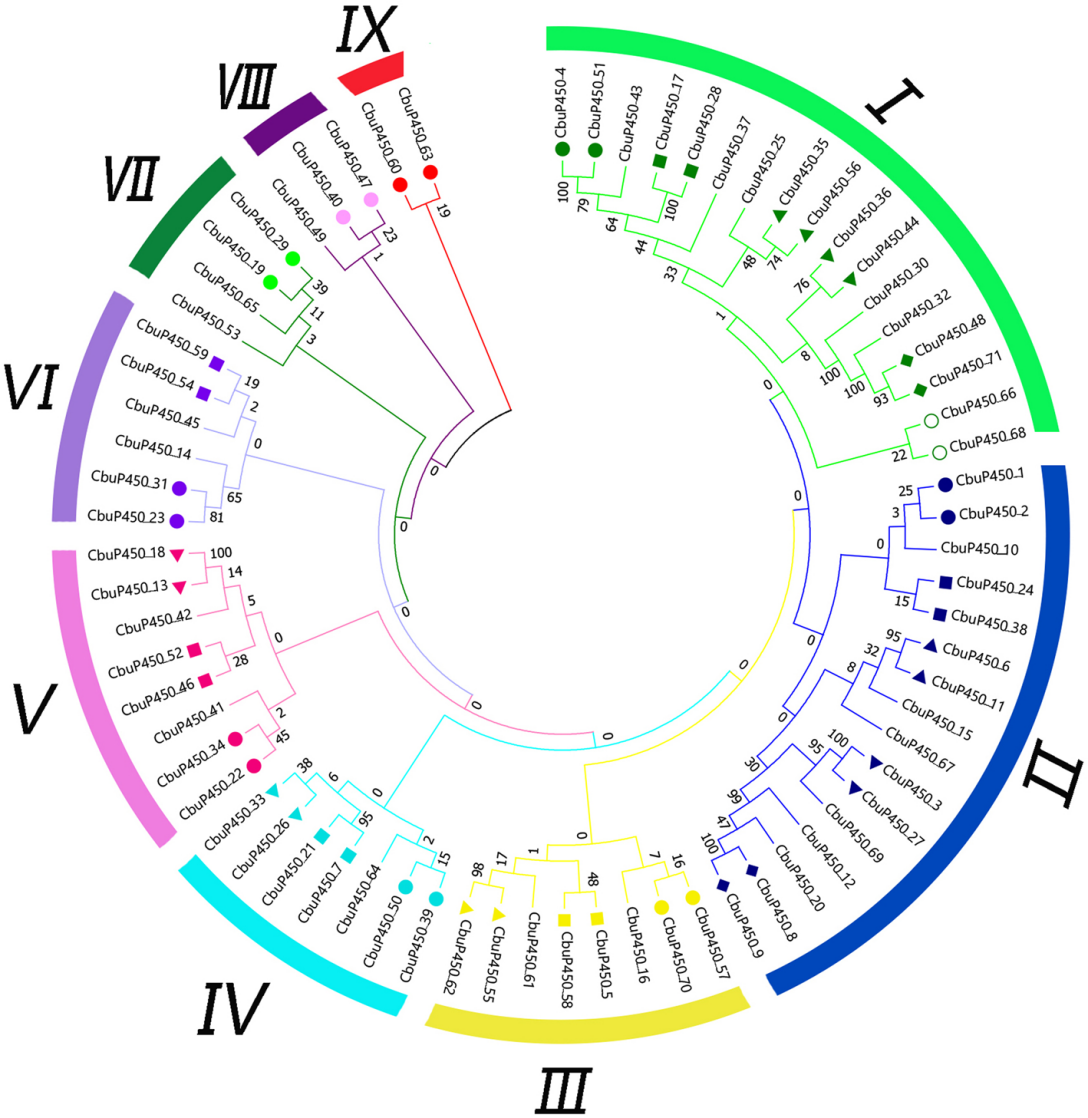
Molecular evolution analysis of P450 protein family in *C. buqueti*

The protein sequences of *CbuP450* gene family were multiplied and analyzed, and the protein sequences of 71 CbuP450 members were entered into the MEGA to map out the evolutionary tree of CbuP450 proteins. From the clustering results of the evolutionary tree, it can be seen that these 71 CbuP450 proteins are divided into 9 groups (called I, II, III, IV, V, VI, VII, VIII, IX). Among them, the I group has the largest number of CbuP450 members, with 17, namely CbuP450_4, CbuP450_51, CbuP450_43, CbuP450_17, CbuP450_28, CbuP450_37, CbuP450_25, CbuP450_35, CbuP450_56, CbuP450_36, CbuP450_44, CbuP450_30, CbuP450_32, CbuP450_48, CbuP450_71, CbuP450_66 and CbuP450_68, accounting for about 23.9% of the total. The second group, which has 16 CbuP450 members; the III to VIII groups has 8, 7, 8, 6, 4, and 3 CbuP450 members, respectively; the minimum is that the IX group has only 2 two members, CbuP450_60 and CbuP450_63, respectively. From the branching relationship of the evolutionary tree, it can be concluded that the groups I, II, III, IV, V, VI, VII, and VIII are clustered as a large group, indicating that they are more closely related and the IX group is more distantly related to them, so it is speculated that the members of the IX group may be very conserved in the long-term genetic process of the species and the most similar to the ancestral relationship (Fig. 2).

**Fig. 3:**
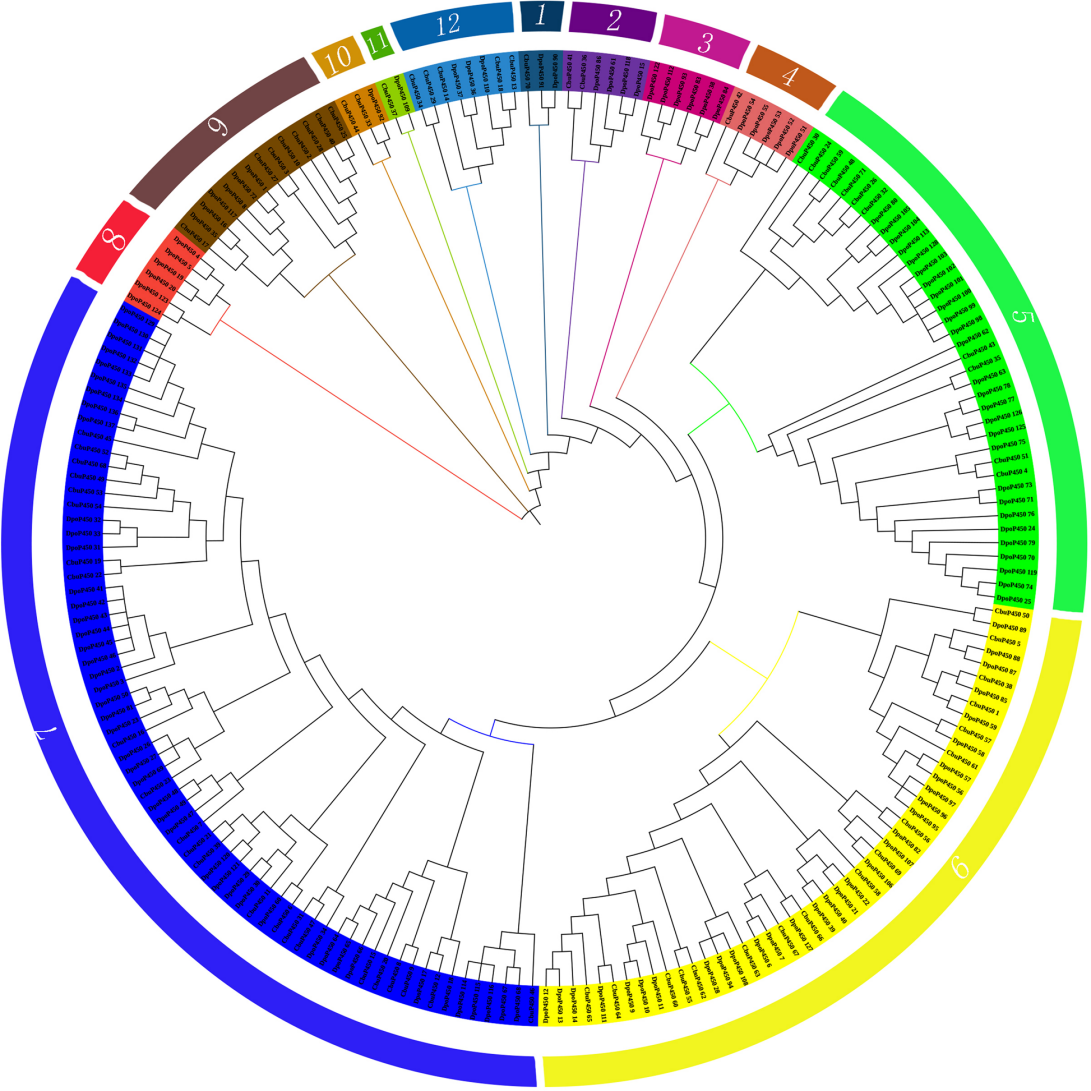
Molecular evolution analysis of P450 protein family between *C. buqueti *and *Dendroctonus ponderosae*

**Fig. 4:**
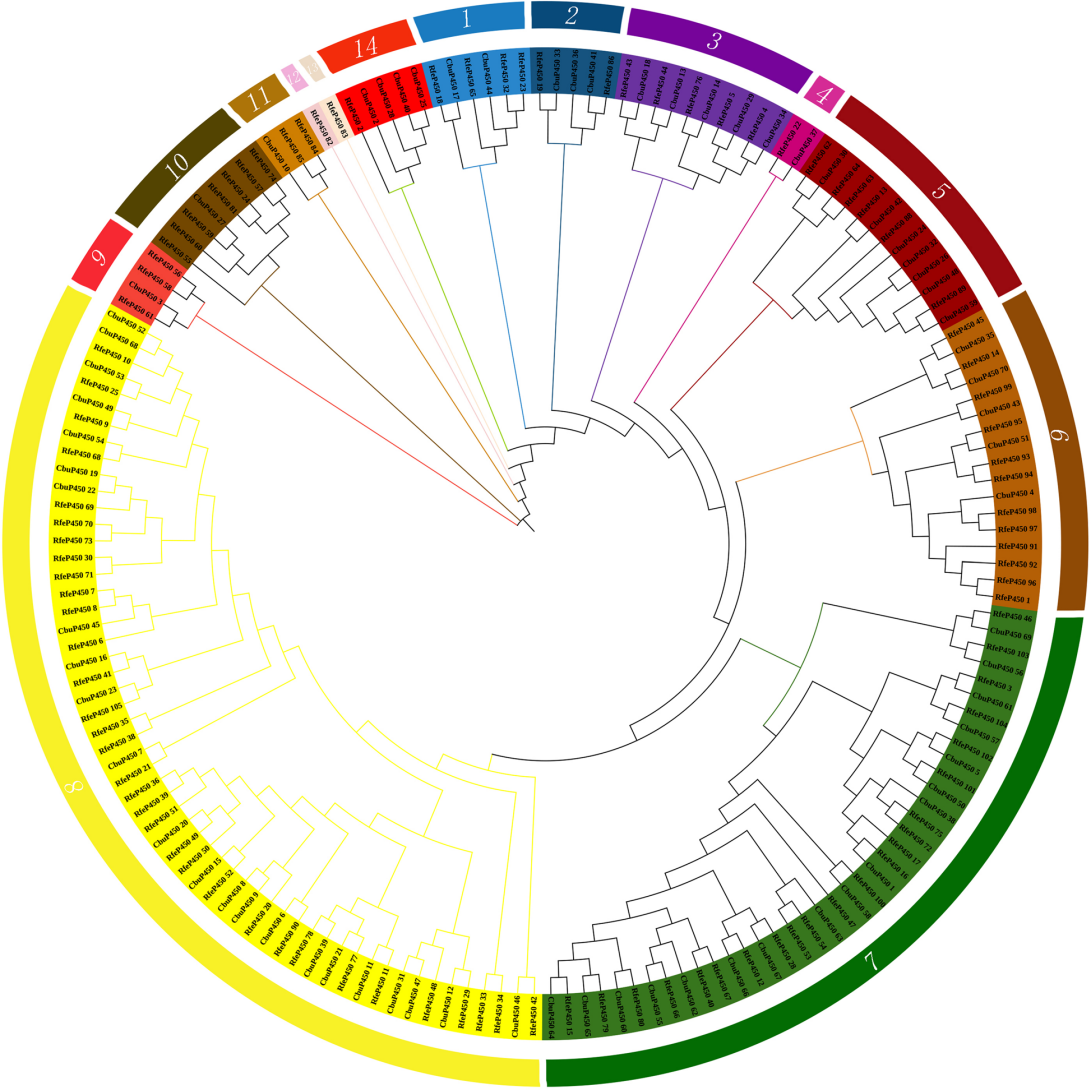
Molecular evolution analysis of P450 protein family between *C. buqueti and Rhynchophorus ferrugineus*

According to the evolutionary trees of the *C. buqueti* and the *Dendroctonus ponderosae*(DpoP450), there are 12 groups, of which 14 pairs of P450 proteins are in the same branch, including CbuP450_14 and DpoP450_82, CbuP450_39 and DpoP450_79, CbuP450_5 and DpoP450_88 etc (Fig. 3). According to the evolutionary trees of *C. buqueti* and *Rhynchophorus ferrugineus*, the P450 family members of the two species were divided into 14 groups, with a total of 44 pairs of P450 proteins in the same small branch, including CbuP450_46 and RfeP450_42, CbuP450_52 and RfeP450_10, CbuP450_14 and RfeP450_76 (Fig. 4). This further indicates that the *C. buqueti* is more closely related to *Rhynchophorus ferrugineus*. Both are weevils, both have a certain degree of damage to plants, and there are certain similarities in the way of destruction, so people also have similarities in their control.

CbuP450:P450 gene name of C. buqueti, DpoP450: P450 gene name of *Dendroctonus ponderosae*

#### CbuP450:P450 protein name of *C. Buqueti*, RfeP450: P450 protein name of *Rhynchophorus Ferrugineus*

**Fig. 5:**
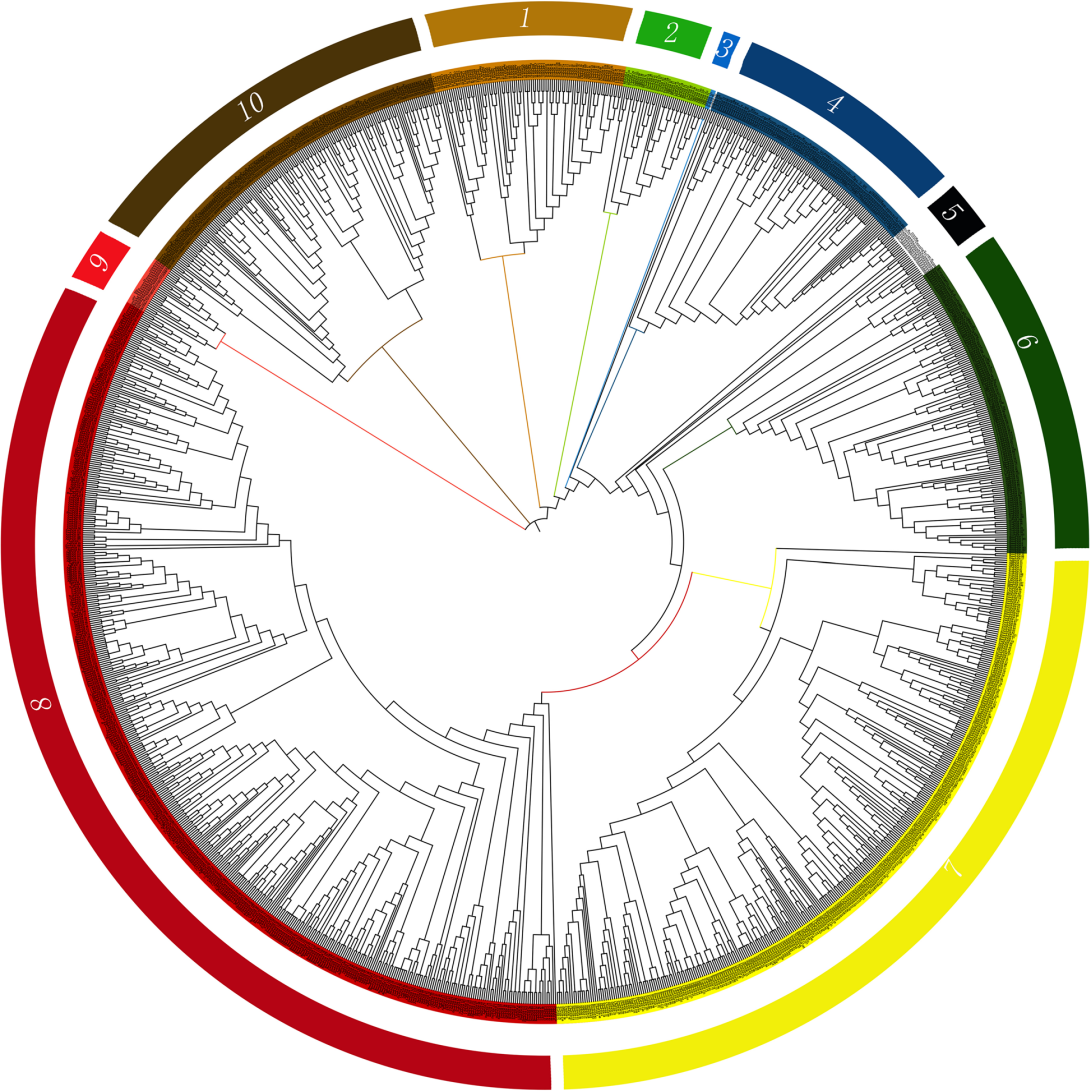
Molecular evolution analysis of P450 protein family between *C. buqueti* and other representative insects

In order to further analyze the affinity and evolutionary pattern of *P450* gene in *C. buqueti*, P450 protein sequences of species such as. *Tribolium castaneum* and *Dendroctonus ponderosae* were selected for homologous clustering analysis. According to the evolutionary analysis of Fig. 5, the evolutionary tree of P450 proteins in *C. buqueti* and other representative insects is divided into 10 groups with other species, of which the 8th group is the most followed by the 7th group, and the 3rd group is the least, with only TcaP450_44 and TcaP450_121. Group 9 is the furthest away, from which it is assumed that the group members are the most conservative. According to the evolutionary analysis of Fig. 3, the *P450* genes of *C. buqueti* and *Dendroctonus ponderosae* are divided into 12 groups, of which the 7th group is the most, and the 11th group has only CbuP450_37 and DpoP450_109. The members of Group 8 contain only *Dendroctonus ponderosaes*, namely DpoP450_4, DpoP450_5, DpoP450_19, DpoP450_20, DpoP450_1223, DpoP450_124, and are the most conservative group. The *P450* members of the *C. buqueti* and *Rhynchophorus ferrugineus* are divided into 14 groups, of which the 8th group is the most, and the 12th and 13th groups have only one member, RfeP450_82, RfeP450_83 respectively. The most conservative is Group 9, which has members RfeP450_56, RfeP450_58, RfeP450_61, CbuP450_3. and *C. buqueti* is more closely related to the *Dendroctonus ponderosae* according to the branching situation, but compared to *Rhynchophorus ferrugineus*, *C. buqueti* is most closely related to the latter (Fig. 5).

### High-frequency codon analysis of *CbuP450* gene family

There are 64 codons in the eukaryotic genome, and these 64 codons encode 20 different amino acids and 3 stop codons, and all amino acids except methionine (Met) and tryptophan (Trp) are encoded by more than one codon. Different codons encoding the same amino acid are synonymous with each other, and different synonymous codons are used at different frequencies, and the frequently used codons are “preferred codons”, while other codons are “non-preferred codons”, a phenomenon called “codon preference”. It is believed that codon bias is caused by the different abundance of tRNAs corresponding to different codons in cells. In general, the higher the abundance of tRNA, the more frequently its corresponding codon will be used.

**Fig. 6:**
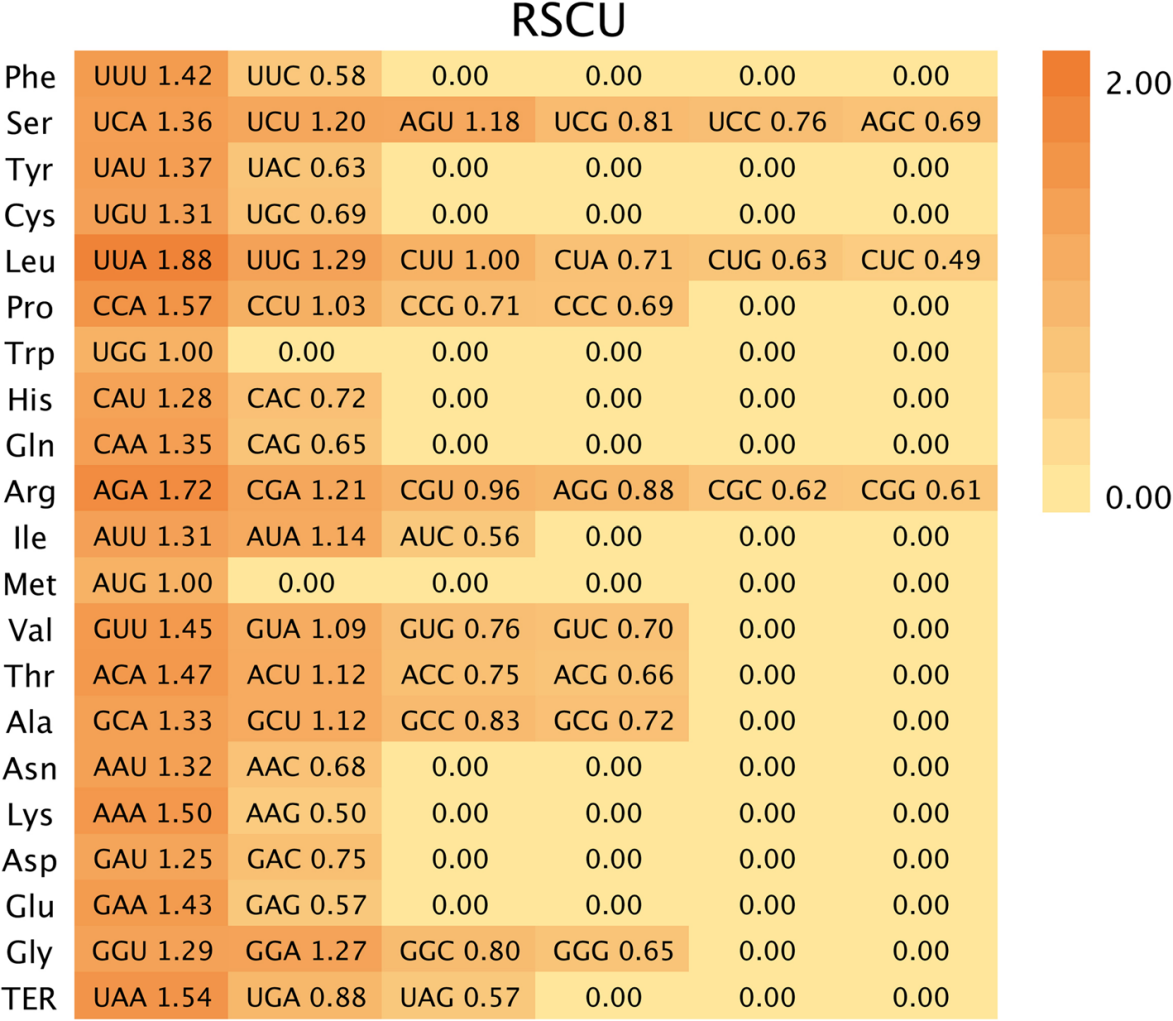
High-frequency codon analysis of *CbuP450* gene family

RSCU value refers to the ratio of the actual frequency of use of a codon to its theoretical expected frequency of use, which is often used as an important parameter to measure the preference of codons. When RSCU = 1, it means that the codon is used at the same frequency as its synonymous codon, and there is no preference. When RSCU > 1, it indicates that its codon use preference is strong, that is, it is considered to be a high-frequency codon. When RSCU < 1, it means that the preference of this codon is weaker than that of other synonymous codons. Among the 71 members of *CbuP450* gene family, there were 29 high-frequency codons, i.e., RSCU value > 1, of which 14 ended in U, 14 ended in A, 1 ended in G, and did not end in C, indicating that the codons ending in U or A were the preferred codons of *CbuP450* gene family, and the codons ending in G or C were non-preferred codons. There are three codons with RSCU value of 1, namely the CUU of methionine, tryptophan and leucine, indicating that there is no preference for the use of these three amino acid codons. The results of RSCU analysis of each amino acid are shown in (Fig. 6).

### Analysis of codon-related parameters of *CbuP450* gene family

Synonymous codon anomalies are usually manifested in codon position 3. In the CbuP450 gene family, the average nucleotide base contents of codon 3 with A, T, C, and G were 43.07%, 41.68%, 22.05%, and 23.01%, respectively. The average GC3s and GC contents were 33.33% and 35.65%, respectively, and the average GC content was less than 50%, indicating that CbuP450 gene family tended to use A/T bases and A/T ending codons.

ENC is the effective codon, which reflects the degree of codon deviation from random selection, and is an important indicator to reflect the degree of non-equilibrium use preference of synonymous codons. The preference of the low-expression genes contained more rare codons was weaker, and the value of ENC was also larger. When the ENC value ≤ 35, it was considered that there was a very significant preference for codon use, and on the whole, the ENC of *CbuP450* gene family was between 48.58 ~ 58.58, and the average value was 54.42, which was much greater than 35, indicating that the preference was not strong.

**Fig. 7:**
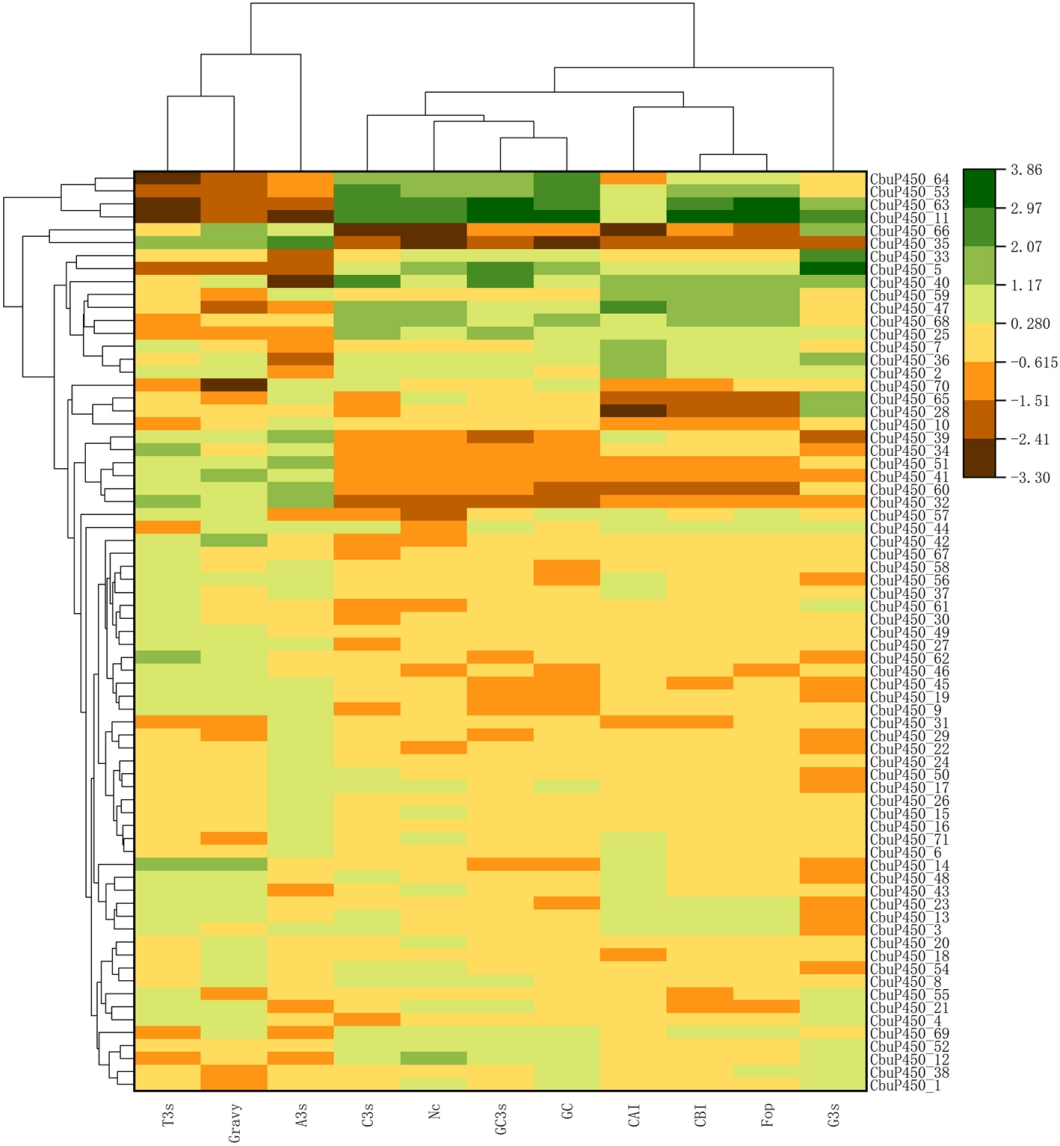
Analysis of codon-related parameters of *P450* gene family in *C. buqueti*

CAI is the codon adaptation index, for a certain gene, CAI refers to the fitness coefficient of all codons encoding the protein relative to the optimal codon of this gene, the CAI value is between 0 ~ 1, the larger the value, the stronger the adaptability. Fop is the optimal codon use frequency, the optimal codon refers to the codon with the highest frequency used in the highly expressed genes of a certain species, and the index refers to the ratio of the optimal codon to its synonymous codon. The FOP range is between 0 and 1, with 1 indicating that only the optimal codon is used and 0 indicating that no optimal codon is used. However, the CAI in this study was between 0.146 ~ 0.198, and the average value was 0.171, which was much less than 1, and the FOP was between 0.313 ~ 0.422, and the average value was 0.357, which was also less than 1, respectively, so it revealed that the codon preference of *CbuP450* gene family was not strong (Fig. 7).

### Gene expression analysis of *CbuP450* gene family

In this study, three sets of parallel experiments were conducted to study the effect of gene expression on the gene expression of adults and larvae of *C. buqueti* at 25 °C as a reference, and under the same conditions, the effect of temperature change on gene expression was studied. At 25 °C, the average value of larvae was 19.38, and at 4 °C, the average value decreased to 18.69, and the expression of larvae for temperature reduction showed a downward trend, among which the *CbuP450_49* decreased the most, from 259.61 at 25 °C to 191.71 at 4 °C, followed by *CbuP450_51*, from 380.51 at 25 °C to 327.72 at 4 °C; *CbuP450_17*, from 80.93 at 25 °C to 144.96 at 4 °C, followed by *CbuP450_10*, from 38.05 at 25 °C to 60.85 at 4 °C.

For the females in the adult worms, the average value was 15.88 at 25 °C, and the average value increased at 42 °C to 28.60, and the gene expression of females for temperature increase showed an upward trend, among which the *CbuP450_23* increased the most, from 21.39 at 25 °C to 335.92 at 42 °C, followed by *CbuP450_10*, from 70.82 at 25 °C to 329.67 at 42 °C, and the most significant downward adjustment was CbuP450_ 29, from 182.63 at 25 °C to 89.47 at 42 °C, followed by *CbuP450_55*, from 37.65 at 25 °C to 6.51 at 42 °C.

As far as a single gene is concerned, at 25 °C, the expression level of *CbuP450_29* in adult females is the highest, and the average value of the three groups of experiments is 182.63, and when the temperature is raised to 42 °C, the average value is 89.47, which is almost half, at this time, the expression level is the highest in *CbuP450_23*, with an average value of 335.92, but at 25 °C, its expression level is only 21.39, which is the opposite of *CbuP450_29*, showing a significant increase. At 25 °C, the highest expression level of larvae was *CbuP450_49*, with an average of 259.61, and when the temperature increased to 42 °C, the average expression was 191.71, showing a downward trend, and at this time, the average amount of *CbuP450_51* was as high as 327.72, compared with 380.51 at 25 °C, it also showed a downward trend. At 25 °C, *CbuP450_61* only the second group of experiments measured a little expression of 0.149, the other two groups were 0, when the temperature rose to 42 °C, the three groups all measured a certain amount of expression, the average value of *CbuP450_46* was 0.632, a small increase. *CbuP450_28* was not expressed in the three groups of experiments at 25 °C, and the expression level was 0, but when the temperature was increased to 42 °C, the average value of *CbuP450_28* three groups of experiments was increased, and the average value reached 0.160, and the average value of *CbuP450_46* two groups of experiments was improved, and the average value reached 0.031. Only *CbuP450_34* had no gene expression either at 25–42 °C.

**Fig. 8:**
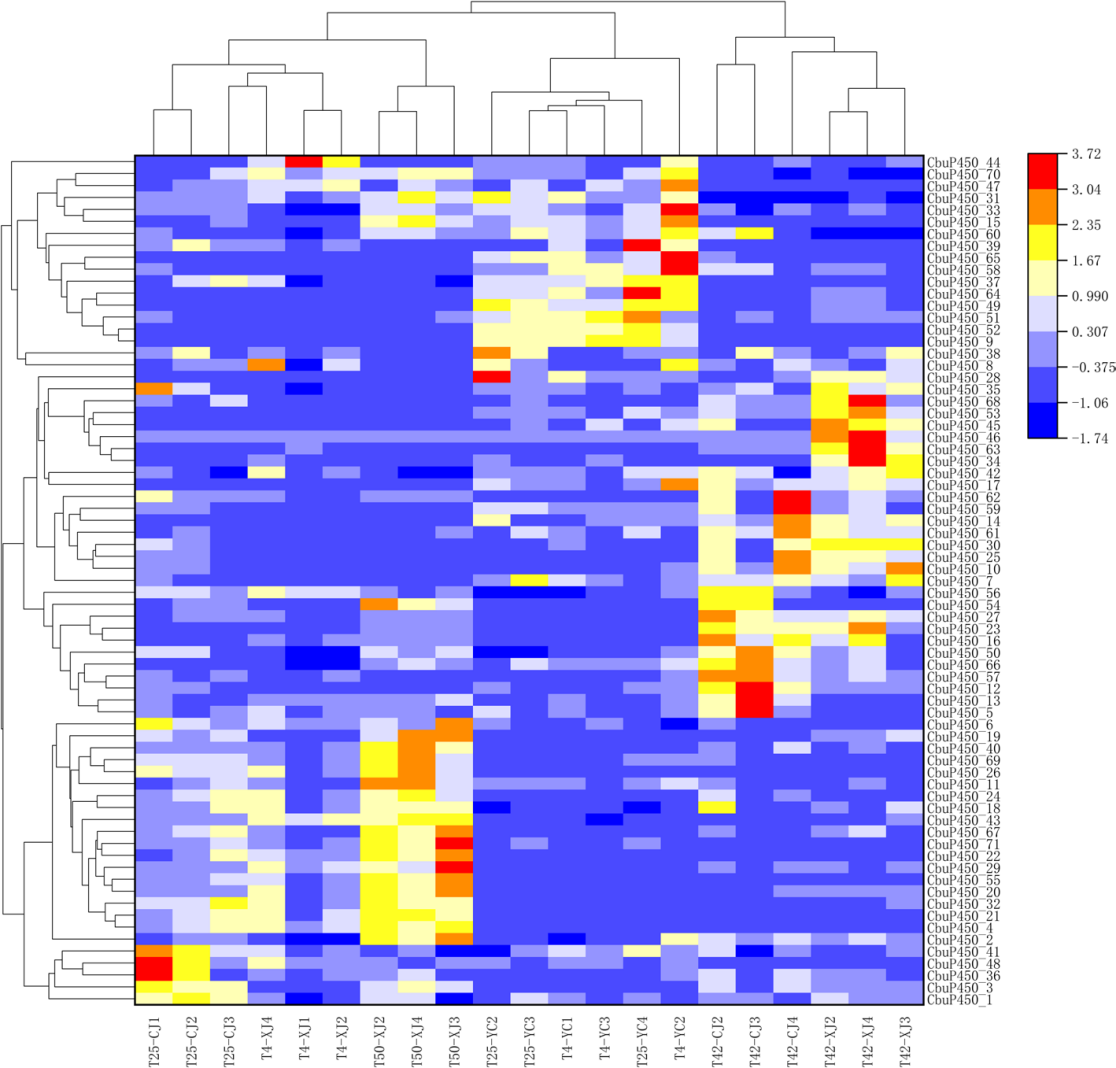
*CbuP450* gene expression analysis at different temperatures

At different temperatures, the gene expression level will change, and the expression level of different genes under the same conditions is also different, and the larvae are more likely to express at 25 °C than the female, so finding the optimal temperature is of great significance for gene expression (Fig. 8).

T25-CJ1:Muscle tissue sample 1 of female adult after treatment at 25 ℃, T25-CJ2:Muscle tissue sample 2 of female adult after treatment at 25 ℃, T25-CJ3:Muscle tissue sample 3 of female adult after treatment at 25 ℃, T4-XJ1:Muscle tissue sample 1 of male adult after treatment at 4 ℃, T4-XJ2:Muscle tissue sample 2 of male adult after treatment at 4 ℃, T4-XJ4:Muscle tissue sample 4 of male adult after treatment at 4 ℃, T50-XJ2:Muscle tissue sample 2 of male adult after treatment at 50 ℃, T50-XJ3:Muscle tissue sample 3 of male adult after treatment at 50 ℃, T50-XJ4:Muscle tissue sample 4 of male adult after treatment at 50 ℃, T25-YC2:Muscle tissue sample 2 of larva after treatment at 25 ℃, T25-YC3:Muscle tissue sample 3 of larva after treatment at 25 ℃, T25-YC4:Muscle tissue sample 4 of larva after treatment at 25 ℃, T4-YC1:Muscle tissue sample 1 of larva after treatment at 4 ℃, T4-YC2:Muscle tissue sample 2 of larva after treatment at 4 ℃, T4-YC3:Muscle tissue sample 3 of larva after treatment at 4 ℃, T42-CJ2:Muscle tissue sample 2 of female adult after treatment at 42 ℃, T42-CJ3:Muscle tissue sample 3 of female adult after treatment at 42 ℃, T42-CJ4:Muscle tissue sample 4 of female adult after treatment at 42 ℃, T42-XJ2:Muscle tissue sample 2 of male adult after treatment at 42 ℃, T42-XJ3:Muscle tissue sample 3 of male adult after treatment at 42 ℃, T42-XJ4:Muscle tissue sample 4 of male adult after treatment at 42 ℃.

Within 2 h after feeding, the transcriptome content of male and female intestinal microorganisms was measured at intervals of 0 h, 0.5 h, 1 h and 2 h, respectively, through three sets of parallel experiments. On the whole, the average expressionmeasured by males was 31.30 at 0 h immediately after eating, and decreased to 27.54 after 0.5 h, increased to 31.31 after 1 h, and decreased to 29.01 after 2 h. After 0.5 h, the average value decreased the most in CbuP450_70, from 158.77 to 37.50, followed by *CbuP450_69*, from 88.81 to 17.73, and the largest increase was in *CbuP450_17*, from 87.92 to 165.86, followed by *CbuP450_25*, from 103.80 to 138.19. The largest decrease in 1 h was *CbuP450_70*, which decreased from 158.77 to 24.77, followed by *CbuP450_69*, which decreased from 88.81 to 14.96, and the largest increase was *CbuP450_23*, which increased from 91.82 to 250.38, followed by CbuP450_16, which rose from 50.62 to 116.44. The largest decrease in 2 h was *CbuP450_70*, which decreased from 158.77 to 48.10, followed by *CbuP450_69*, which decreased from 88.81 to 21.70, and the largest increase was in *CbuP450_25*, which increased from 103.80 to 166.05, followed by *CbuP450_17*, which increased from 165.86 to 135.24.

The average expression of females was 34.41, and the average values increased after 0.5 h, 1 h, and 2 h, which were 34.83, 38.26, and 39.17, respectively. After 0.5 h, the average value decreased the most in *CbuP450_30*, from 197.12 to 120.55, followed by *CbuP450_59*, from 72.39 to 40.66, and the largest increase was in *CbuP450_16*, from 451.82 to 549.28, followed by *CbuP450_23*, from 454.81 to 525.35. The largest decrease in 1 h was *CbuP450_30*, which decreased from 197.12 to 85.36, followed by *CbuP450_51*, which decreased from 101.37 to 54.43, and the largest increase was in *CbuP450_10*, which increased from 286.73 to 2304.69, followed by *CbuP450_16*, which rose from 451.82 to 569.58. The largest decrease in 2 h was *CbuP450_30*, which decreased from 197.12 to 100.83, followed by *CbuP450_59*, which decreased from 72.39 to 40.41, and the largest increase was in *CbuP450_23*, which increased from 454.81 to 687.21, followed by *CbuP450_16*, which increased from 451.82 to 663.50. Overall, the number of transcriptome counts increased more significantly in females than males after a longer period of digestion.

In terms of a single gene, in the first group of experiments of male worms, compared with 0 h, there were 23 members that decreased after 0.5 h, and 2 members remained unchanged, namely *CbuP450_45* and *CbuP450_47*, and the remaining 47 members all increased, 18 decreased after 1 h, and only 1 member remained unchanged, which was *CbuP450_45*, and the remaining 52 members all increased, and 21 members decreased after 2 h, and only *CbuP450_45*, and the remaining 49 members all rose; In the second group, there were 47 members who decreased after 0.5 h, only *CbuP450_39* remained unchanged, the remaining 23 members all increased, 48 members decreased after 1 h, *CbuP450_39* and the remaining 22 members all increased, and the number of members who decreased after 2 h was relatively small, with 39 members, and *CbuP450_39* the remaining 31 members all increased. In the third group, there were 43 members who decreased after 0.5 h, and there were no members who remained unchanged, the remaining 28 members all increased, 45 members decreased after 1 h, and the remaining 26 members all increased, and the number of members who decreased after 2 h was the largest, with a total of 50, and the remaining 21 members all showed an increase.

**Fig. 9:**
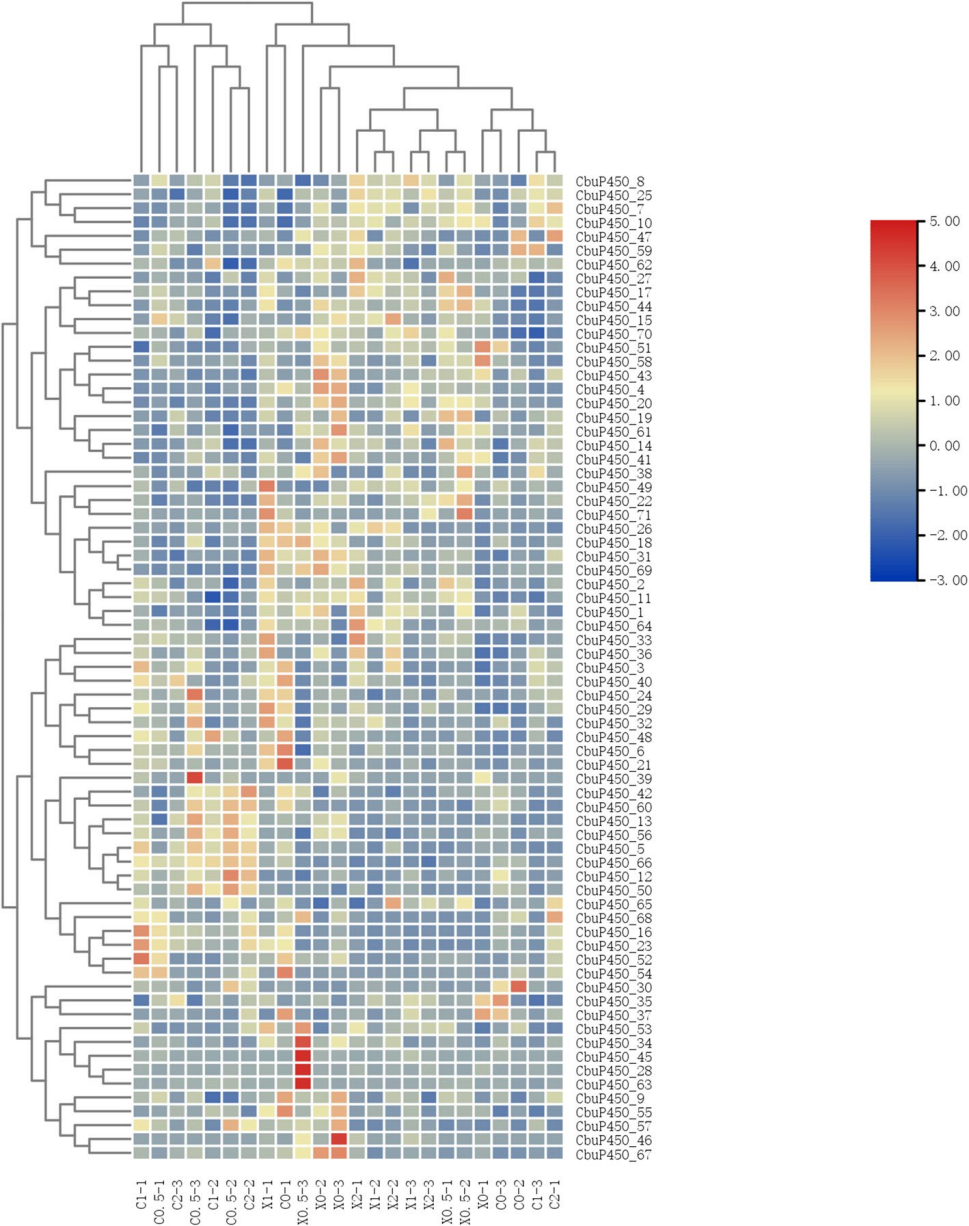
Gene expression level analysis of *CbuP450* genes in transcriptome under different feeding times

In the first group of experiments of females, compared with 0 h, there were 23 members that decreased after 0.5 h, no members remained unchanged, the remaining 48 members all increased, 36 decreased after 1 h, no unchanged members, the remaining 35 members all increased, 51 members decreased after 2 h, no unchanged members, and the remaining 20 members all increased. In the second group, there were 33 members who decreased after 0.5 h, the remaining 23 members all increased, 36 members decreased after 1 h, and 2 members remained unchanged, respectively *CbuP450_39 and CbuP450_57*, and the remaining 33 members all increased, and the number of members who decreased after 2 h was also 33, and only one member remained unchanged *CbuP450_39*. In the third group, there were 22 members who decreased after 0.5 h, and there were no members who remained unchanged, and the remaining 49 members all increased, 43 members who decreased after 1 h, and 2 members who remained unchanged, respectively *CbuP450_39* and *CbuP450_47*, and the remaining 26 members all increased, and the number of members who decreased after 2 h was 33, and only one member remained unchanged was also *CbuP450_39 *(Fig. 9).

C0-1:intestinal contents sample 1 of Female insect without feeding, C0-2:intestinal contents sample 2 of Female insect without feeding, C0-3:intestinal contents sample 3 of Female insect without feeding, C0.5-1:intestinal contents sample 1 of Female insect with feeding for 0.5 h, C0.5-2:intestinal contents sample 2 of Female insect with feeding for 0.5 h, C0.5-3:intestinal contents sample 3 of Female insect with feeding for 0.5 h, C1-1:intestinal contents sample 1 of Female insect with feeding for 1 h, C1-2:intestinal contents sample 2 of Female insect with feeding for 1 h, C1-3:intestinal contents sample 3 of Female insect with feeding for 1 h, C2-1:intestinal contents sample 1 of Female insect with feeding for 2 h, C2-2:intestinal contents sample 2 of Female insect with feeding for 2 h, C2-3:intestinal contents sample 3 of Female insect with feeding for 2 h.

X0-1:intestinal contents sample 1 of male insect without feeding, X0-2:intestinal contents sample 2 of male insect without feeding, X0-3:intestinal contents sample 3 of male insect without feeding, X0.5-1:intestinal contents sample 1 of male insect with feeding for 0.5 h, X0.5-2:intestinal contents sample 2 of male insect with feeding for 0.5 h, X0.5-3:intestinal contents sample 3 of male insect with feeding for 0.5 h, X1-1:intestinal contents sample 1 of male insect with feeding for 1 h, X1-2:intestinal contents sample 2 of male insect with feeding for 1 h, X1-3:intestinal contents sample 3 of male insect with feeding for 1 h, X2-1:intestinal contents sample 1 of male insect with feeding for 2 h, X2-2:intestinal contents sample 2 of male insect with feeding for 2 h, X2-3:intestinal contents sample 3 of male insect with feeding for 2 h.

Within 2 h after eating, the protein content of intestinal microorganisms in the *C. buqueti* was measured at intervals of 0 h, 0.5 h, 1 h, and 2 h, respectively, through three sets of parallel experiments. Through data comparison, it can be seen that the overall protein content is not high, whether it is a female or a male, only 14 members can be measured, which are *CbuP450_1, CbuP450_2, CbuP450_9, CbuP450_10, CbuP450_14, CbuP450_17, CbuP450_18, CbuP450_20, CbuP450_23, CbuP450_25, CbuP450_29, CbuP450_35, CbuP450_49, CbuP450_51*, and the remaining 58 members are all 0.

On the whole, the average protein content of males was 1.045 at 0 h immediately after feeding, and decreased to 0.947 and 0.999 after 0.5 h and 1 h, respectively, and increased to 1.121 after 2 h. After 0.5 h, the average value decreased the most in *CbuP450_17*, from 1.141 to 0.902, followed by *CbuP450_10*, from 1.112 to 0.888, and the largest increase was in *CbuP450_23*, from 0.883 to 1.124, followed by *CbuP450_29*, from 0.982 to 1.124. The largest decrease in 1 h was in *CbuP450_20*, which decreased from 1.168 to 0.960, followed by *CbuP450_17*, which decreased from 1.141 to 0.974, and the largest increase was in *CbuP450_23*, which rose from 0.883 to 0.983, followed by *CbuP450_9*, which rose from 0.976 to 1.075. The largest decrease in 2 h was *CbuP450_17*, which decreased from 1.141 to 1.065, followed by *CbuP450_49*, which decreased from 1.032 to 0.973, and the largest increase was in *CbuP450_23*, which rose from 0.883 to 0.987, followed by *CbuP450_29*, which rose from 0.982 to 1.036.

The average protein content of females was 0.965, which increased after 0.5 h, 1 h and 2 h, which were 1,019, 0.990 and 1.017, respectively. After 0.5 h, the average value decreased the most in *CbuP450_10*, from 0.934 to 0.841, followed by *CbuP450_14*, which decreased from 0.956 to 0.908, and the largest increase was in *CbuP450_29*, which increased from 0.987 to 1.181, followed by *CbuP450_18*, which increased from 0.933 to 1.118. The largest decrease in 1 h was in *CbuP450_23*, which decreased from 1.033 to 0.838, followed by *CbuP450_9*, which decreased from 1.065 to 0.95, and the largest increase was in CbuP450_25, which rose from 0.877 to 1.074, followed by *CbuP450_35*, which rose from 0.919 to 0.940. The largest decrease in 2 h was in *CbuP450_49*, which decreased from 1.001 to 0.944, followed by *CbuP450_23*, which decreased from 1.033 to 0.984, and the largest increase was in *CbuP450_14*, which rose from 0.956 to 1.138, followed by *CbuP450_25*, which rose from 0.877 to 1.025.

In terms of a single gene, the highest protein content was 1.258 in *CbuP450_20*, 1.202 in the second *CbuP450_49* group, and 1.373 in the third group *CbuP450_29* the highest in the first group after eating. After 0.5 h, the highest protein content was *CbuP450_17* in the first and second groups, which were 1.087 and 1.425, respectively, and the highest protein content in the third group was *CbuP450_20*, which was 1.458. At this time, if only the 13 groups of members whose protein content can be measured are compared, the 5 members in the first group who decreased were *CbuP450_9, CbuP450_18, CbuP450_20, CbuP450_29, CbuP450_49* and *CbuP450_51*, and 7 members who increased, namely *CbuP450_2, CbuP450_10, CbuP450_14, CbuP450_17, CbuP450_23, CbuP450_25, CbuP450_35* and 3 members of the second group declined, namely *CbuP450_9, CbuP450_18* and *CbuP450_49*, and the remaining 10 members all increased. In the third group, 4 members declined, namely *CbuP450_2, CbuP450_17, CbuP450_18 and CbuP450_29*, and the remaining 9 members all increased. In the first group of females, the highest protein content was also measured immediately after eating, with *CbuP450_49* (1.276), the second group had the highest *CbuP450_9* content (1.163), the third group had the highest *CbuP450_18* (1.14), and the highest protein content was *CbuP450_23* in the first and second groups after 0.5 h, which were 1.43 and 1.168, respectively, and the highest in the third group was *CbuP450_29*, which was 1.318. At this time, if only the 13 groups of members whose protein content could be measured were compared, the 5 members in the first group decreased *CbuP450_2, CbuP450_9*, *CbuP450_20, CbuP450_49* and *CbuP450_51*, and the remaining 8 members all increased. In the second group, 4 members declined, namely *CbuP450_2, CbuP450_9, CbuP450_14 and CbuP450_18*, while the remaining 9 members all increased. There were 4 members in the third group, namely *CbuP450_20, CbuP450_35, CbuP450_49 and CbuP450_51*, and the remaining 9 members all increased.

After 1 h, the highest *CbuP450_10* content in the first group was 1.213, 1.112 in the second group of *CbuP450_14*, and 1.242 in the third group *CbuP450_25*. In the first group, there were 6 members who rose, namely *CbuP450_10, CbuP450_14,CbuP450_23, CbuP450_25, CbuP450_29*, and *CbuP450_35*, and 7 members who fell. In the second group, there were 7 members who rose, namely *CbuP450_2, CbuP450_9, CbuP450_14, CbuP450_23, CbuP450_29, CbuP450_35* and *CbuP450_51*, and the remaining 6 members all increased. In the third group, 9 members rose, and the remaining 4 members all declined, namely *CbuP450_17, CbuP450_18, CbuP450_35* and *CbuP450_49*. The highest *CbuP450_9* content was 1.231 in the first group of females, 1.278 in the second group of *CbuP450_25*, and 1.263 in the third group CbuP450_2. There were 5 members in the first group who declined, namely *CbuP450_2, CbuP450_20, CbuP450_23, CbuP450_49* and *CbuP450_51*, and the remaining 8 members all increased, and 7 members in the second group decreased, namely *CbuP450_2, CbuP450_9, CbuP450_20, CbuP450_23, CbuP450_29, CbuP450_49, CbuP450_51*, the remaining 6 members all increased, and in the second group, 4 members declined, namely *CbuP450_17, CbuP450_18, CbuP450_35 and CbuP450_49*, and the remaining 9 members all increased.

**Fig. 10:**
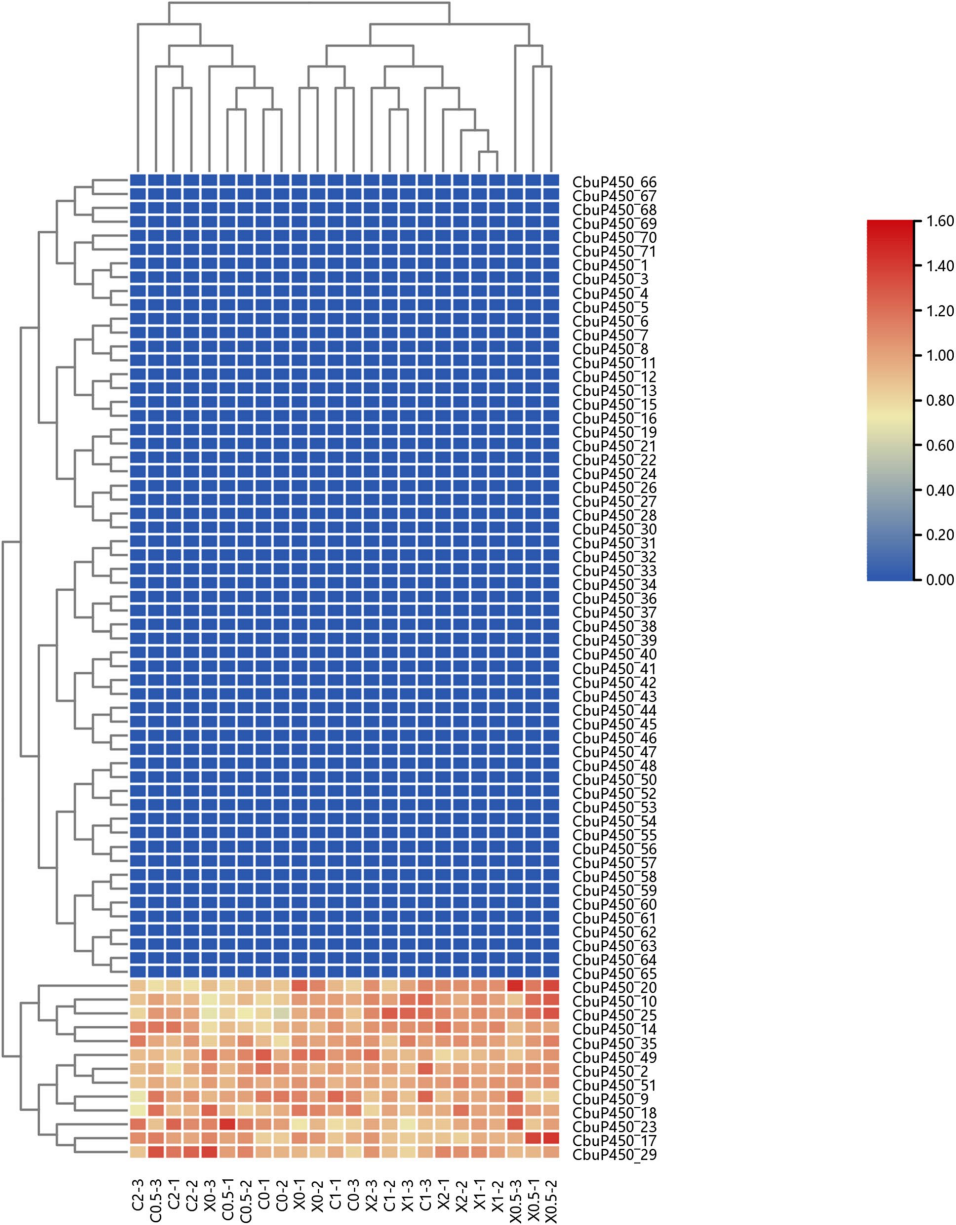
10 CbuP450 protein content analysis under different feeding times

After 2 h, the highest CbuP450_14 content in the first group was 1.206, 1.192 in the second group of *CbuP450_18*, and 1.201 in the third group *CbuP450_49*. In the first group, there were 5 members who rose, namely *CbuP450_14, CbuP450_23, CbuP450_25, CbuP450_29* and *CbuP450_35*, and the remaining 8 members all declined. In the second group, there were 7 members who rose, namely *CbuP450_2, CbuP450_14, CbuP450_18, CbuP450_23, CbuP450_29, CbuP450_35* and *CbuP450_51*, and the remaining 6 members all declined. In the third group, there were 6 members who rose, namely *CbuP450_10, CbuP450_14, CbuP450_20, CbuP450_29, CbuP450_35* and *CbuP450_49*, and the remaining 7 members all rose. Among the females, the highest in the first and third groups were *CbuP450_23*, with 1.247 and 1.186, respectively, and the highest in the second group was *CbuP450_29*, with 1.25. In the first group, there were 6 members who rose, namely *CbuP450_10, CbuP450_14, CbuP450_17, CbuP450_23, CbuP450_25* and *CbuP450_29*, and the remaining 7 members all increased. In the second group, there were 7 members who rose, namely *CbuP450_10, CbuP450_14, CbuP450_17, CbuP450_23, CbuP450_25, CbuP450_29* and *CbuP450_35*, and the remaining 6 members all increased. In the third group, 6 members rose, namely *CbuP450_14, CbuP450_17, CbuP450_20, CbuP450_23, CbuP450_29* and *CbuP450_35*, and the remaining 7 members all rose. Both females and males have higher gut microbial protein content after a longer period of digestion (Fig. 10).

C0-1:intestinal contents sample 1 of Female insect without feeding, C0-2:intestinal contents sample 2 of Female insect without feeding, C0-3:intestinal contents sample 3 of Female insect without feeding, C0.5-1:intestinal contents sample 1 of Female insect with feeding for 0.5 h, C0.5-2:intestinal contents sample 2 of Female insect with feeding for 0.5 h, C0.5-3:intestinal contents sample 3 of Female insect with feeding for 0.5 h, C1-1:intestinal contents sample 1 of Female insect with feeding for 1 h, C1-2:intestinal contents sample 2 of Female insect with feeding for 1 h, C1-3:intestinal contents sample 3 of Female insect with feeding for 1 h, C2-1:intestinal contents sample 1 of Female insect with feeding for 2 h, C2-2:intestinal contents sample 2 of Female insect with feeding for 2 h, C2-3:intestinal contents sample 3 of Female insect with feeding for 2 h.

X0-1:intestinal contents sample 1 of male insect without feeding, X0-2:intestinal contents sample 2 of male insect without feeding, X0-3:intestinal contents sample 3 of male insect without feeding, X0.5-1:intestinal contents sample 1 of male insect with feeding for 0.5 h, X0.5-2:intestinal contents sample 2 of male insect with feeding for 0.5 h, X0.5-3:intestinal contents sample 3 of male insect with feeding for 0.5 h, X1-1:intestinal contents sample 1 of male insect with feeding for 1 h, X1-2:intestinal contents sample 2 of male insect with feeding for 1 h, X1-3:intestinal contents sample 3 of male insect with feeding for 1 h, X2-1:intestinal contents sample 1 of male insect with feeding for 2 h, X2-2:intestinal contents sample 2 of male insect with feeding for 2 h, X2-3:intestinal contents sample 3 of male insect with feeding for 2 h.

### Protein-protein interaction analysis of CbuP450 protein family

**Fig. 11:**
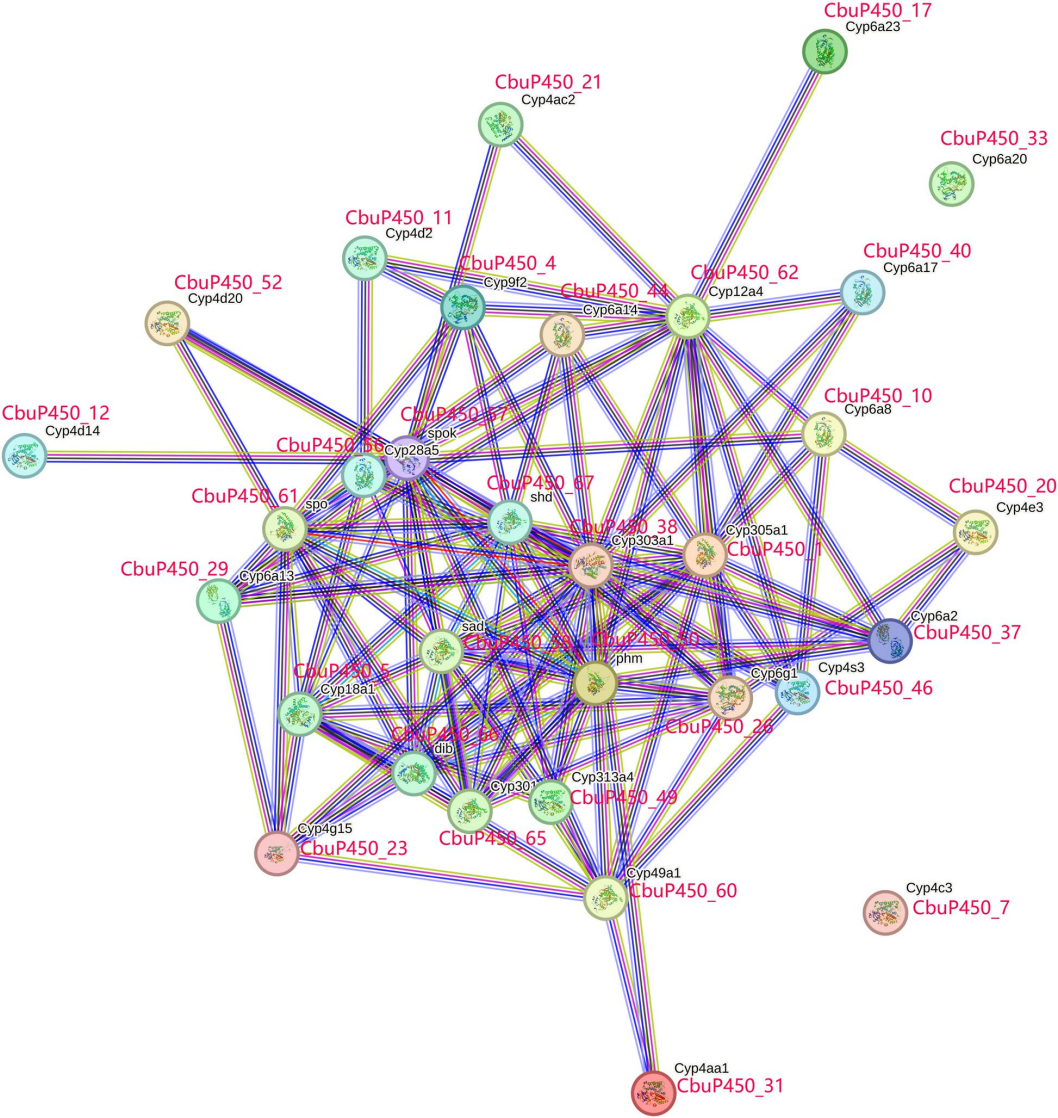
Protein-protein interaction analysis of CbuP450 protein family

By constructing the interaction relationship between 71 CbuP450 proteins and referring to *Drosophila melanogaster*, the analysis results showed that among 71 CbuP450 proteins, two protein members were independent, namely CbuP450_33 and CbuP450_31, indicating that these two protein members had no interaction relationship with other proteins. Most protein members have more than one interaction, for example, CbuP450_11 interact with CbuP450_56, CbuP450_41, CbuP450_62, etc., indicating that they have direct or indirect regulatory roles with each other. By referring to Drosophila proteins, among the proteins involved in interactions, there are mainly the following types: SAD (CbuP450_58), which encodes cytochrome P450 involved in ecdyssteroid biosynthesis. It shows mitochondrial localization and catalyzes the addition of hydroxyl groups to the 2 carbons of the cholesterol ring. Its mutants cannot experience head degeneration, dorsal closure, or secrete the stratum corneum. At the same time, PHM (CbuP450_50), SHD (CbuP450_67), spok (CbuP450_57), spo (CbuP450_61), Cyp18a1 (CbuP450_5), dib (CbuP450_66) and other cytochrome P450 are also biosynthesized by ecdysteroids. Cyp6g1 (CbuP450_26), Cyp4s3 (CbuP450_46), Cyp6a2 (CbuP450_37), Cyp6a8 (CbuP450_10), Cyp305a1 (CbuP450_1), Cyp12a4 (CbuP450_62), Cyp6a23 (CbuP450_17), Cyp6ac14 (CbuP450_44), Cyp4ac2 (CbuP450_21), Cyp9f2 (CbuP450_4), Cyp4d2 (CbuP450_11), Cyp4d20 (CbuP450_52), Cyp4d14 (CbuP450_12), Cyp6a13 (CbuP450_29), Cyp313a4 (CbuP450_49), Cyp49a1 (CbuP450_60), Cyp4aa1 (CbuP450_31) and other involved in the decomposition of synthetic insecticides, which may be related to the metabolism of insect hormones. Cyp301a1 (CbuP450_49) encodes a member of the cytochrome P450 family that has both mitochondrial and cytosolic roles in the detoxification of compounds (Fig. 11).

### qRT-PCR validation analysis of *CbuP450* gene expression under different conditions

The gene expression analysis of *P450* gene family of *C. buqueti* at different temperatures showed that there were completely non-expressing members at 25 °C, which were *CbuP450_28, CbuP450_34*, and *CbuP450_46* in females and *CbuP450_19, CbuP450_26*, and *CbuP450_57* in larvae. The highest expression in females was *CbuP450_28*, and the highest expression in larvae was *CbuP450_49*.

**Fig. 12:**
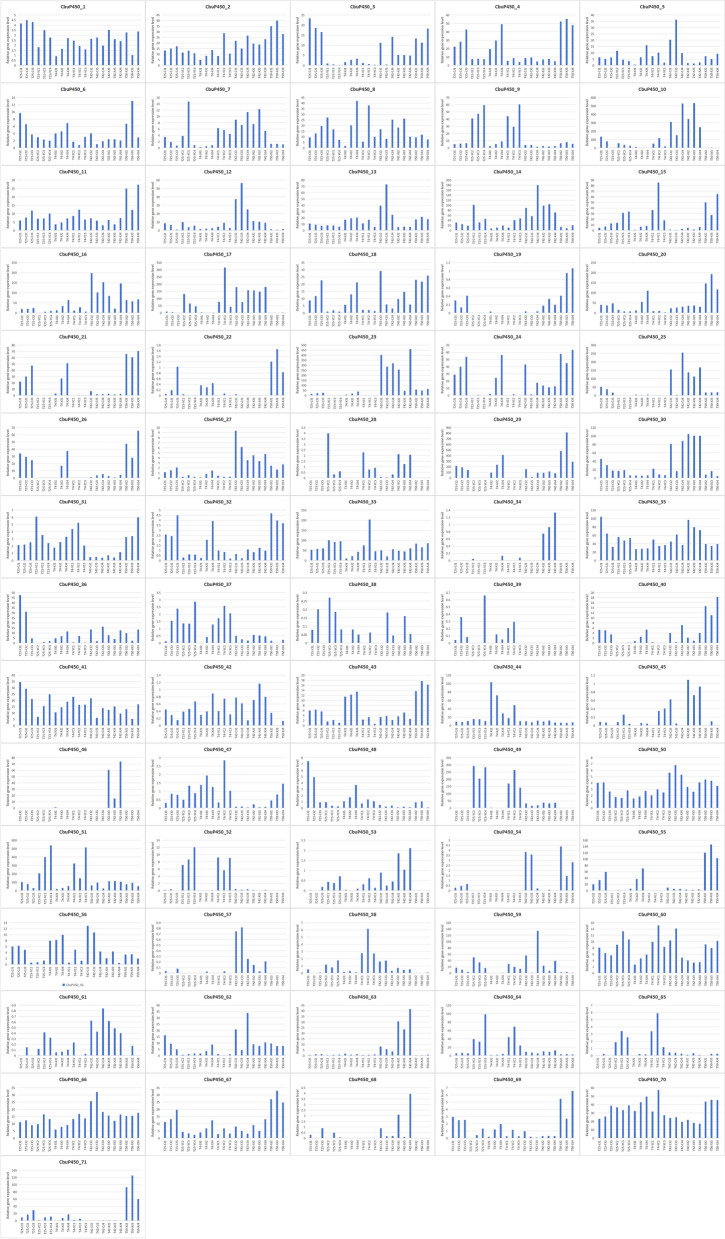
qRT-PCR verification analysis of *CbuP450* gene expression at different temperatures

When the temperature rose to 42 °C, the gene expression of 34 *CbuP450* genes in the female worm increased, including *CbuP450_1, CbuP450_3, CbuP450_4, CbuP450_5, CbuP450_6, CbuP450_7, CbuP450_9 ~ CbuP450_24, CbuP450_26, CbuP450_28, CbuP450_29, CbuP450_31, CbuP450_32, CbuP450_33, CbuP450_35, CbuP450_36, CbuP450_37, CbuP450_38, CbuP450_39, CbuP450_40, CbuP450_41, CbuP450_43, CbuP450_47, CbuP450_48, CbuP450_49, CbuP450_55, CbuP450_50, CbuP450_51, CbuP450_53, CbuP450_58, CbuP450_64, CbuP450_65, CbuP450_66, CbuP450_67, CbuP450_69, CbuP450_70, CbuP450_71*, among which the *CbuP450_23* reaches the maximum value and rises the most, which may play an important role in high temperature resistance; There are 36 *CbuP450* genes who have declined, namely *CbuP450_2, CbuP450_5, CbuP450_7, CbuP450_8, CbuP450_10, CbuP450_12 ~ CbuP450_14, CbuP450_16, CbuP450_17, CbuP450_23, CbuP450_25, CbuP450_27, CbuP450_28, CbuP450_30, CbuP450_42, CbuP450_44 ~ CbuP450_46, CbuP450_49, CbuP450_50, CbuP450_52 ~ CbuP450_54, CbuP450_56 ~ CbuP450_66, CbuP450_68.* Among them, *CbuP450_21* decreases the most, and may not be resistant to high temperatures. At the same time, there are also members who do not express at 42 °C, namely *CbuP450_39* and *CbuP450_34*, which are speculated to be not resistant to high temperatures. When the temperature dropped to 4 °C, 31 *CbuP450* genes of the larvae fell, including *CbuP450_1, CbuP450_4, CbuP450_6, CbuP450_7, CbuP450_9, CbuP450_12, CbuP450_14, CbuP450_20, CbuP450_25 ~ CbuP450_28, CbuP450_30, CbuP450_31, CbuP450_35, CbuP450_38, CbuP450_39, CbuP450_42, CbuP450_46, CbuP450_49, CbuP450_51 ~ CbuP450_53, CbuP450_55, CbuP450_59, CbuP450_61, CbuP450_62, CbuP450_64, CbuP450_68, CbuP450_69, CbuP450_ 71*, of which, the *CbuP450_49* decreased the most, and it is speculated that it may not be resistant to low temperatures; There were 38 *CbuP450* genes whose gene expression increased, including *CbuP450_2, CbuP450_3, CbuP450_5, CbuP450_8, CbuP450_10, CbuP450_11, CbuP450_13, CbuP450_15, CbuP450_16, CbuP450_17, CbuP450_18, CbuP450_21 ~ CbuP450_24, and CbuP450_29, CbuP450_32 ~ CbuP450_34, CbuP450_36, CbuP450_37, CbuP450_40, CbuP450_41, CbuP450_43 ~ CbuP450_45, CbuP450_47, CbuP450_48, CbuP450_50, CbuP450_56 ~ CbuP450_58, CbuP450_60, CbuP450_63, CbuP450_65 ~ CbuP450_67 and CbuP450_70*, among which the *CbuP450_17* rises the most, and it is speculated that it may be resistant to low temperatures; At the same time, there are three members who do not express themselves, including *CbuP450_54, CbuP450_68*, and *CbuP450_19*, and may not be resistant to low temperatures (Fig. 12).

T25-CJ1:Muscle tissue sample 1 of female adult after treatment at 25 ℃, T25-CJ2:Muscle tissue sample 2 of female adult after treatment at 25 ℃, T25-CJ3:Muscle tissue sample 3 of female adult after treatment at 25 ℃, T4-XJ1:Muscle tissue sample 1 of male adult after treatment at 4 ℃, T4-XJ2:Muscle tissue sample 2 of male adult after treatment at 4 ℃, T4-XJ4:Muscle tissue sample 4 of male adult after treatment at 4 ℃, T50-XJ2:Muscle tissue sample 2 of male adult after treatment at 50 ℃, T50-XJ3:Muscle tissue sample 3 of male adult after treatment at 50 ℃, T50-XJ4:Muscle tissue sample 4 of male adult after treatment at 50 ℃, T25-YC2:Muscle tissue sample 2 of larva after treatment at 25 ℃, T25-YC3:Muscle tissue sample 3 of larva after treatment at 25 ℃, T25-YC4:Muscle tissue sample 4 of larva after treatment at 25 ℃, T4-YC1:Muscle tissue sample 1 of larva after treatment at 4 ℃, T4-YC2:Muscle tissue sample 2 of larva after treatment at 4 ℃, T4-YC3:Muscle tissue sample 3 of larva after treatment at 4 ℃, T42-CJ2:Muscle tissue sample 2 of female adult after treatment at 42 ℃, T42-CJ3:Muscle tissue sample 3 of female adult after treatment at 42 ℃, T42-CJ4:Muscle tissue sample 4 of female adult after treatment at 42 ℃, T42-XJ2:Muscle tissue sample 2 of male adult after treatment at 42 ℃, T42-XJ3:Muscle tissue sample 3 of male adult after treatment at 42 ℃, T42-XJ4:Muscle tissue sample 4 of male adult after treatment at 42 ℃.

The gene expression analysis of *P450* gene family in *C. buqueti* after different feeding times was verified by qRT-PCR experiment. After 0.5 h, there were 32 members with increased expression values in males, respectively *CbuP450_1, CbuP450_3, CbuP450_7, CbuP450_8, CbuP450_9, CbuP450_10, CbuP450_11, CbuP450_12, CbuP450_14, CbuP450_17, CbuP450_18, CbuP450_19, CbuP450_20, CbuP450_22, CbuP450_25, CbuP450_27 ~ CbuP450_31, CbuP450_33, CbuP450_38, CbuP450_40, CbuP450_44, CbuP450_46, CbuP450_49, CbuP450_50, CbuP450_52, CbuP450_59, CbuP450_60, CbuP450_6.* Among them, the largest increase was in *CbuP450_17*, and the remaining 39 *CbuP450* genes all declined, of which *CbuP450_70* decreased the most. There were 36 *CbuP450* genes with increased expression values in females, respectively *CbuP450_5, CbuP450_7, CbuP450_8, CbuP450_10, CbuP450_12 ~ CbuP450_17, CbuP450_23, CbuP450_24, CbuP450_27 ~ CbuP450_29, CbuP450_32, CbuP450_33, CbuP450_36, CbuP450_38, CbuP450_39, CbuP450_41, CbuP450_44, CbuP450_45, CbuP450_46, CbuP450_48, CbuP450_50, CbuP450_56, CbuP450_57, CbuP450_58, CbuP450_60, CbuP450_63, CbuP450_65, CbuP450_66, CbuP450_67, CbuP450_70.* Among them, *CbuP450_16* increased the most, and the remaining 35 members all declined, of which the *CbuP450_30* decreased the most.

After 1 h, there were 36 *CbuP450* genes with increased expression values in males, which were respectively *CbuP450_1, CbuP450_2, CbuP450_3, CbuP450_6, CbuP450_8, CbuP450_11, CbuP450_16, CbuP450_17, CbuP450_18, CbuP450_19, CbuP450_21 ~ CbuP450_26, CbuP450_28 ~ CbuP450_31, CbuP450_33, CbuP450_36, CbuP450_40, CbuP450_46 ~ CbuP450_50, CbuP450_53, CbuP450_56, CbuP450_59, CbuP450_60, CbuP450_64, CbuP450_66, CbuP450_67, CbuP450_71.* Among them, the largest increase was in *CbuP450_23*, and the remaining 35 *CbuP450* genes all declined, of which *CbuP450_70* decreased the most. There were 30 *CbuP450* genes with increased expression values in females, respectively *CbuP450_2, CbuP450_3, CbuP450_5, CbuP450_7, CbuP450_8, CbuP450_10, CbuP450_12, CbuP450_13, CbuP450_14, CbuP450_16, CbuP450_23, CbuP450_25, CbuP450_28, CbuP450_29, CbuP450_33, CbuP450_36, CbuP450_38, CbuP450_39, CbuP450_40, CbuP450_41, CbuP450_48, CbuP450_50, CbuP450_52, CbuP450_56, CbuP450_57, CbuP450_59, CbuP450_61, CbuP450_62, CbuP450_66, CbuP450_67 Among them, the CbuP450_10* increased the most, and the remaining 41 *CbuP450* genes all declined, of which *CbuP450_30* decreased the most.

**Fig. 13:**
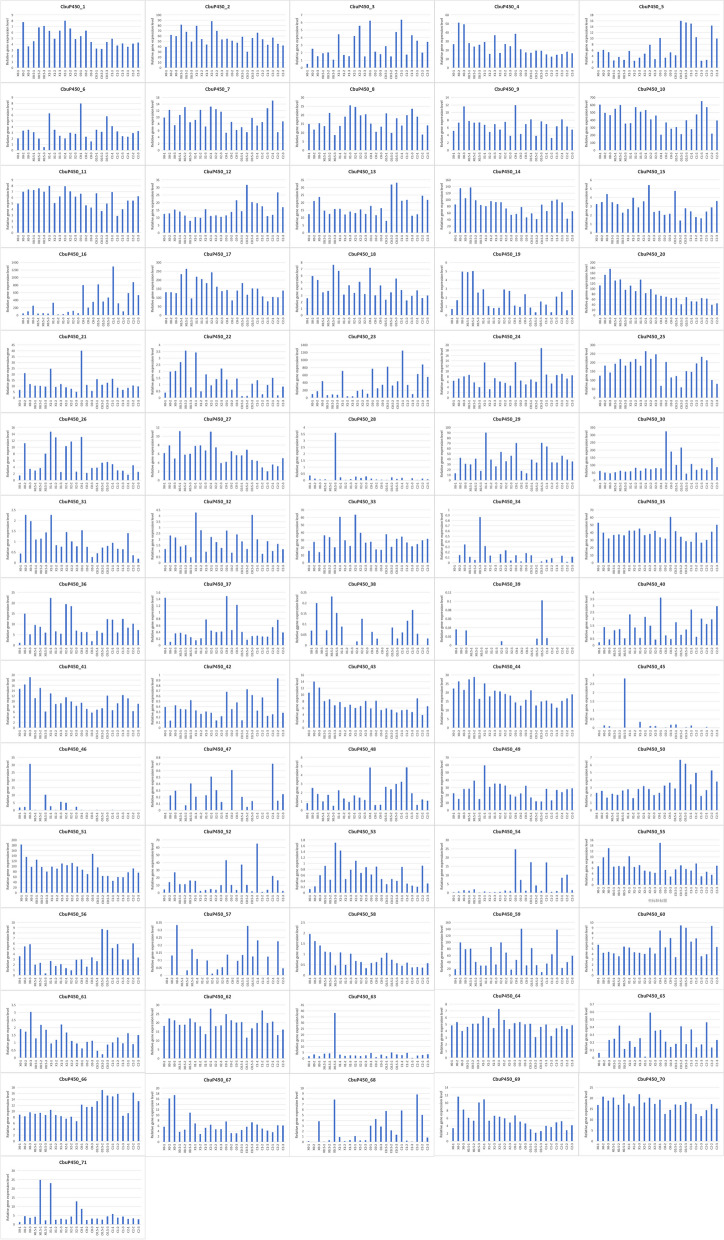
qRT-PCR verification analysis of CbuP450 gene expression after different feeding times

After 2 h, there were 32 *CbuP450* genes with increased expression value in males, respectively *CbuP450_1, CbuP450_2, CbuP450_3, CbuP450_5, CbuP450_7, CbuP450_8, CbuP450_9, CbuP450_11, CbuP450_15 ~ CbuP450_19, CbuP450_23, CbuP450_25 ~ CbuP450_30, CbuP450_33, CbuP450_36, CbuP450_38, CbuP450_46, CbuP450_47, CbuP450_49, CbuP450_58, CbuP450_59, CbuP450_60, CbuP450_64, CbuP450_66, CbuP450_67.* Among them, *CbuP450_25* increased the most, and the remaining 39 *CbuP450* genes all declined, of which *CbuP450_70* decreased the most. There were 30 *CbuP450* genes with increased expression values in females, respectively *CbuP450_5, CbuP450_7, CbuP450_8, CbuP450_10 ~ CbuP450_16, CbuP450_19, CbuP450_23, CbuP450_25, CbuP450_29, CbuP450_33, CbuP450_36, CbuP450_40, CbuP450_41, CbuP450_44, CbuP450_47, CbuP450_49, CbuP450_50, CbuP450_56, CbuP450_57, CbuP450_61, CbuP450_65, CbuP450_66, CbuP450_67, CbuP450_68, CbuP450_70.* Among them, the largest increase was in *CbuP450_23*, and the remaining 41 *CbuP450* genes all declined, of which the largest decline was in *CbuP450_30*. It was speculated that the *CbuP450_17, CbuP450_23* and *CbuP450_25* in males and the *CbuP450_10, CbuP450_16* and *CbuP450_23* in females may be related to the regulation of bamboo fiber degradation genes in *C. buqueti* (Fig. 13).

C0-1:intestinal contents sample 1 of Female insect without feeding, C0-2:intestinal contents sample 2 of Female insect without feeding, C0-3:intestinal contents sample 3 of Female insect without feeding, C0.5-1:intestinal contents sample 1 of Female insect with feeding for 0.5 h, C0.5-2:intestinal contents sample 2 of Female insect with feeding for 0.5 h, C0.5-3:intestinal contents sample 3 of Female insect with feeding for 0.5 h, C1-1:intestinal contents sample 1 of Female insect with feeding for 1 h, C1-2:intestinal contents sample 2 of Female insect with feeding for 1 h, C1-3:intestinal contents sample 3 of Female insect with feeding for 1 h, C2-1:intestinal contents sample 1 of Female insect with feeding for 2 h, C2-2:intestinal contents sample 2 of Female insect with feeding for 2 h, C2-3:intestinal contents sample 3 of Female insect with feeding for 2 h.

X0-1:intestinal contents sample 1 of male insect without feeding, X0-2:intestinal contents sample 2 of male insect without feeding, X0-3:intestinal contents sample 3 of male insect without feeding, X0.5-1:intestinal contents sample 1 of male insect with feeding for 0.5 h, X0.5-2:intestinal contents sample 2 of male insect with feeding for 0.5 h, X0.5-3:intestinal contents sample 3 of male insect with feeding for 0.5 h, X1-1:intestinal contents sample 1 of male insect with feeding for 1 h, X1-2:intestinal contents sample 2 of male insect with feeding for 1 h, X1-3:intestinal contents sample 3 of male insect with feeding for 1 h, X2-1:intestinal contents sample 1 of male insect with feeding for 2 h, X2-2:intestinal contents sample 2 of male insect with feeding for 2 h, X2-3:intestinal contents sample 3 of male insect with feeding for 2 h.

## Discussion

After a detailed bioinformatics analysis of P450 protein members in *C. buqueti*, the various characteristics of P450 protein members of *C. buqueti* can be understood in more detail [24]. *C. buqueti* contain 71 *CbuP450* genes [23], and 31 *P450* genes were screened from the saltwater flea genome, and 77 *P450* genes were found in the wild silkworm genome. It can be seen that the number of P450 protein members in different species is inconsistent. The protein family members of *C. buqueti* contain 183 to 1041 amino acids, contain multiple conserved structures, there are signal peptides and transmembrane domain structures, and the average hydrophilicity of the P450 protein is mostly less than 0, so it is a hydrophilic protein. Its isoelectric point is between 5.93 and 9.41, the isoelectric point of the oriental bee is 8.67 [25], the isoelectric point of the small brown planthopper is about 5.37 [26], and the isoelectric point of the large diamond flat fish is 5.75 [27], and the amino acid isoelectric point of these insect P450 protein family members is almost in the same range as the isoelectric point of the *P450* in *C. buqueti*. In the analysis of secondary structure, the component content of the secondary structure is α-helix > random coil > extension chain > β-turn angle from large to small, and the α-spiral and random coil are also the majority in temperate bed bugs. Seven of P450 protein families have signal peptides, and only one of the N-terminals of beet armyworms [28], but none of them have signal peptides in the *Aedes aegypti* mosquitoes [29], indicating that there are secreted proteins in the P450 protein family of the long-lived *C. buqueti*, and the other two substances do not contain secreted proteins, which are synthesized by free ribosomes, proteins that enter cytosols, and participate in the chemical reactions of mitochondria, nuclei, and peroxidase bodies.

Among insects, following Ray’s discovery of the presence of *P450* in houseflies in 1967, the presence of *P450* was found in dozens of insects such as tobacco moths, locusts, Anopheles mosquitoes, etc. Wu Yidong et al., a scientist who has studied, found that the distribution of *P450* in different tissues of the sixth-instar larvae is significantly different, with the highest content in the midgut, followed by fat and body weight, and the lowest in the body wall [30]. Lepidoptera is second only to Coleoptera, and also occupies an important position in insects, according to studies, the number of cytochrome *P450* genes varies greatly among different Lepidoptera insects, among which the gene function related to insect growth and development is relatively conservative and the number is basically constant; and the gene function related to resistance metabolism is related to the special living environment and feeding habits of insects, and the number varies greatly, so it is speculated that the *C. buqueti* also has similar characteristics [31]. The secondary structure of cytochrome *P450* of *Aedes aegyptis* [29] and *Plasmodium falciparum* [32] consists mainly of α spirals and irregular curls, the same as that of *C. buqueti*, presumably because the cytochrome *P450* of different insects has a similar folding structure. There are 71 *P450* genes in *C. buqueti*, which is relatively small compared to all other animals, such as the 483 *P450* genes of *Mansoni vampires* [33]. The reason for this may be that the study of *C. buqueti* lags behind the study of other animals, so there are other new *P450*s to supplement. The distribution of the *P450* gene family and its evolutionary laws of *C. buqueti* are of great value in the development and application of biological control. In this study, it was found that the *P450* family protein of the *C. buqueti* has phosphorylation sites at serine, complexine and threonine, and it is speculated that the protein is regulated by phosphorylation to achieve its function. After analysis, the presence of transmembrane regions of *C. buqueti* is relatively large, with vampire having an N-terminal transmembrane region [33], while neither *Aedes aegypti* [29] nor *Plasmodium falciparum* [32] indicates that the first two may belong to membrane proteins and the latter two are not. According to the branching situation, *C. buqueti* and *Dendroctonus ponderosae* are more closely related, but compared with the red-brown weevil, *C. buqueti* is more closely related to the latter. The red palm weevil is a pest that is seriously harmful to palm plants [34] ,whose larvae feed on the main stem cause irreversible damage to the host plant and severely cause plant death in the short term [5] Studies have shown that it is the most destructive pest of palm trees worldwide, and functional analysis has demonstrated that the up-regulation of *P450* gene expression has evolved into adaptation to insecticide stress caused by exposure to the systemic neonicotinoid insecticide imidacloprid [35]. *C. buqueti* is most closely related to *Rhynchophorus ferrugineus*, and it is speculated that the large *C. buqueti* has the same relationship with insecticides. According to amino acid domain analysis, most of 71 *CbuP450* genes have Motif1 to Motif10. Similarly, on the amino acid multi-sequence alignment of *Hatz trichoderma* cytochrome *P450* family genes that respond to pine wood nematode stress, the 10 CYP genes also have multiple conserved sites that form conserved domains [36]. Gene conservatism usually confers similar, basic, and indispensable functions to life, and there is no doubt about its importance, which may be why most genes have most conserved structures. The genetic structure analysis of *P450* family in *C. buqueti* shows that 16 *CbuP450* genes have 7 exons and 6 introns, exactly the same as Mansonite, it also has 7 exons and 6 introns [33], and the vast majority of the substances contain exons and introns, possibly due to the reduction of the damage of gene mutations to the coding sequence.According to the chromosome localization analysis results, 71 *CbuP450* genes in *C. buqueti* are unevenly distributed on 11 chromosomes, of which Chr2 has the largest number of genes, with 19. In other insect studies, chromosome localization analysis has not been found for the time being, but 226 *P450* genes have been found and preliminarily identified in the genome of pineapple, and 226 genes are distributed on 17 chromosomes through gene localization analysis, of which except for the distribution of gene clusters on chromosomes 4, 5 and 9, other chromosomes have gene cluster distribution. It is speculated that *P450* gene family is more commonly distributed on chromosomes, and it is easier to find that it does not occur only in a specific chromosome. Among the 71 members of this gene family, there are 29 high-frequency codons, which tend to use A/T bases and A/T ending codons, ENC is between 48.58 ~ 58.58, the average value is 54.42, CAI is between 0.146 ~ 0.198, the average value is 0.171, FOP is between 0.313 ~ 0.422, and the average value is 0.357, which is also less than 1, and the codon preference is not strong. The ENC range of hedgehogs was 31.83 ~ 50.67, with an average value of 43.37, and the preference ended in base A or U(T) [37], and species of the Drosophila subgenus showed reduced codon use bias, and likewise a reduced preference for codons ending in C, but the codon use patterns of all species except for G in the second position were influenced by the 2nd and 3rd nucleotides of the codon, rather than the biochemical properties of the encoded amino acids [37]. At 25 °C, the larvae of *P450* gene family were more likely to be expressed than the females, and the female Cbu*P450*_23 may play an important role in high temperature resistance, and the larval *CbuP450_17* may play an important role in low temperature tolerance, but the larvae have a downward trend in gene expression for temperature reduction, and the gene expression of female insect for temperature increase has an upward trend. Brooding at 30 °C can help chicks gain weight, develop small intestine morphology, improve immunity, and express genes related to intestinal nutrient absorption [38]. Low temperatures of 19 °C and below can induce reversal from females to males [39], and among the four Arabidopsis LNKs, LNK1 and LNK2 function under normal and high temperature conditions, and LNK3 and LNK4 work under cold conditions. Thus, these LNK proteins play an important role in the induction of specific genes under different temperature conditions [40]. In both animals and plants, temperature is important for growth, developmental timing, and sex determination/differentiation. Gene expression analysis was performed at different feeding times (0 h, 0.5 h, 1 h, 2 h), and both females and males had higher intestinal microbial protein content after digestion for a longer time, but the transcriptome FKPM values of females increased more significantly than those of males, and *CbuP450_10, CbuP450_17*, and *CbuP450_23* in males and *CbuP450_10, CbuP450_16* and in females *CbuP450_20, CbuP450_23* and *CbuP450_29* may be related to the regulation of bamboo fiber degradation genes by the *C. buqueti*. By constructing the interaction relationship of 71 proteins of *P450* gene family in *C. buqueti* and referring to Drosophila protein, it was found that two protein members were independent, namely CbuP450_33 and CbuP450_31, indicating that there was no interaction between these two protein members and other proteins, and most of the protein members had more than one interaction relationship, and among the proteins participating in the interaction, they were mainly divided into SAD (CbuP450_58) and PHM (CbuP450_50) It is involved in the biosynthesis of ecdyssteroids, Cyp6g1 (CbuP450_26) and Cyp4s3 (CbuP450_46) are involved in the decomposition of synthetic insecticides and the metabolism of insect hormones, and Cyp301a1 (CbuP450_49) is involved in the detoxification of compounds. At the same time, it has been found that cytochrome *P450* superfamily genes that are highly expressed in the larval stage of *Anopheles sinensis* may be involved in the detoxification of foodborne toxic compounds in the larval stage, and cytochrome *P450* genes that are highly expressed in the midgut and marmobitian ducts of female adult mosquitoes may play an important role in the detoxification process [41].

## Conclusions

In this study, we have identified 71 *CbuP450* genes in *C. buqueti* genome. We systematically analyzed the gene family by using bioinformatics methods. The analysis results showed that CbuP450 proteins are proteins with transmembrane domains. 71 *CbuP450* genes are unevenly distributed across 11 chromosomes, and all genes contain introns. *C. buqueti* and *Rhynchophorus ferrugineus* were the most closely related. This gene family has 29 high-frequency codons, which tend to use A/T bases and A/T ending codons. *CbuP450_23* in the female adult may play an important role on high temperature resistance, and Cbu*P450*_17 in the larval may play an important role on low temperature tolerance. *CbuP450_10, CbuP450_17, CbuP450_23, CbuP450_10, CbuP450_16, CbuP450_20, CbuP450_23* and *CbuP450_29* may be related to the regulation of bamboo fiber degradation genes in *C. buqueti*. Most CbuP450 proteins are mainly divided into three aspects: encoding the biosynthesis of ecdysteroids, participating in the decomposition of synthetic insecticides, metabolizing insect hormones, and participating in the detoxification of compounds. These results provided a reference for further research on the function of *P450* gene family in *C. buqueti*.

## Materials and methods

### Experimental design and data acquisition

The adults and larval samples of *C. buqueti* used in this study were collected in Danan Town, Muchuan County, Leshan City, Sichuan Province, and the adults and larvae of *C. buqueti* were used for genome sequencing, transcriptome sequencing and proteome sequencing, respectively. Adults (female and male) and larvae of *C. buqueti* were treated at different temperature (25 °C in the control group, 4 °C, 42 °C and 50 °C in the treatment group) and different feeding times (no feeding in the control group (0 h), 0.5 h after feeding fresh bamboo shoots, 1 h after feeding, and 2 h after feeding in the treatment group) were all carried out in the Molecular Biology Laboratory of Key Laboratory of Sichuan Province for Bamboo Pests Control and Resource Development of Leshan Normal University from July 15 to August 15, 2020. All the whole genome sequences, protein sequences, gene annotation files, transcriptome sequencing data of adults and larvae of *C. buqueti* under different temperature conditions, and transcriptome sequencing and proteome sequencing data of *C. buqueti* at different feeding times were downloaded from these data (NCBI genome and transcriptome accession number: PRJNA675312, PRJNA719467 and PRJNA718062) submitted to the NCBI database by Chun Fu et al [23]. The total RNA was extracted from muscle tissue samples of *C. buqueti* under different temperature treatments and intestinal contents samples of *C. buqueti* under different time conditions after feeding. Reverse transcription of purified RNA into cDNA using a reverse transcription kit, and reverse transcribed cDNA was used for qRT-PCR to verify the expression of *CbuP450* genes in *C. buqueti* under different temperature treatments and different time conditions after feeding. The qRT-PCR experiments in this study were all completed on fluorescence quantitative PCR instrument (qToWer3 G) of the Analytick Jena AG. The number of replicates of biological samples in each treatment group and control group is 2, and the number of machine replicates on fluorescence quantitative PCR is 3.

### Identification and physicochemical properties analysis of CbuP450 proteins

The identification method for *P450* gene family in the whole genome of *C. buqueti* is based on the Hmmsearch method using the Pfam model(PF00067) of *P450* gene family and the SMART search method for its conserved structural domains. Only sequences that contain both Pfam models of *P450* and conserved structural domains are true members of *CbuP450* gene family, which eliminates pseudogenes. The physicochemical properties of CbuP450 proteins were analyzed using the PratoParam online program, including the relevant predictions of protein length, molecular weight, isomorphic electricity, instability index, fat coefficient, total number of positive (negative) electrical residues, and total average hydrophilic coefficient [42], and subcellular localization of 71 protein members through the online program Cell-PLoc2.0 [43]. The online software SignalP-3.0 server was used to predict signal peptide of P450 protein family in *C. buqueti* [44].

### Transmembrane structure, hydrophobicity, phosphorylation site and conservative motif analysis of CbuP450 proteins

Enter CbuP450 protein sequences into the TMHMM Server v.2.0 online software to analyze the transmembrane structure of CbuP450 proteins [45]. Its hydrophobicity analysis was done through the Protscale online website [46]. NetPhos3.1 Server was used to predict its potential phosphorylation sites [47]. MEME online tool was used to search for conserved motifs of CbuP450 proteins, and the parameter settings were 10 to maintain the number of base orders, and the other parameters were the default settings [48].

### Secondary structure and tertiary structure analysis of CbuP450 proteins

The SOPMA online website was used to predict secondary structures of CbuP450 proteins. The SWISS-MODEL online tool was used to predict its tertiary structures [49].

### Gene structure, chromosomal localization and codon preference analysis of *CbuP450* gene family

The GSDS2.0 analysis website was used to analyze the genetic structure of all members of *CbuP450* gene family, and the Mapchart tool was used to analyze the chromosome localization of all members of *CbuP450* gene family. CodonW and EMBOSS were used to analyze codon-related parameters and high-frequency codons for *CbuP450* gene family.

### Molecular evolutionary analysis of *CbuP450* gene family

MEGA 7.0 software was used to multi-compare protein sequences and construct an evolutionary tree using the NJ method, with a calibration parameter of 1000 [50].

### Transcriptome expression and qRT-PCR validation analysis of *CbuP450* gene family

Using the reverse transcription kit purchased from Beijing Tiangen Biotech Co., Ltd., the extracted and purified mRNA samples were reverse transcribed into cDNA. With the cDNA as a template, the 18 S rRNA gene as an internal reference gene, and the designed qRT-PCR primers as a guide, the cDNA was subjected to PCR amplification under different temperature treatment conditions and different feeding time treatment conditions to obtain the expression levels of each *CbuP450* gene. The relative expression level of the target gene *CbuP450* was calculated by using the expression level of the reference gene as a reference. TBtools was used to process and heat map the obtained relative expression level of *CbuP450* genes [51]. All *CbuP450* gene primers designed by TBtools Batch q-RT-PCR primer design tool used in the qRT-PCR validation experiment in this study are shown in Supplementary Table 5. The qRT-PCR primers used in this study were synthesized by Sangon Biotech (Shanghai) Co., Ltd on commission.

### Supplementary Information

Supplementary Material 1.

## Data Availability

The genome assembly data and RNA-seq data used in this study were retrieved from the NCBI Sequence Read Archive (SRA) database (https://www.ncbi.nlm.nih.gov/sra) under the accession code PRJNA675312, PRJNA719467 and PRJNA718062.
