# Supplementary material for: Genome-wide identification, molecular evolution and gene expression of P450 gene family in Cyrtotrachelus buqueti
Source: BMC Genomics. 2024 May 8;25:453. doi: 10.1186/s12864-024-10372-5 (PMC11080265; doi:10.1186/s12864-024-10372-5)
Supplement: Supplementary file 1 — Supplementary Material 1. [file 12864_2024_10372_MOESM1_ESM.zip › Supplementary file/Supplementary Figure.docx]

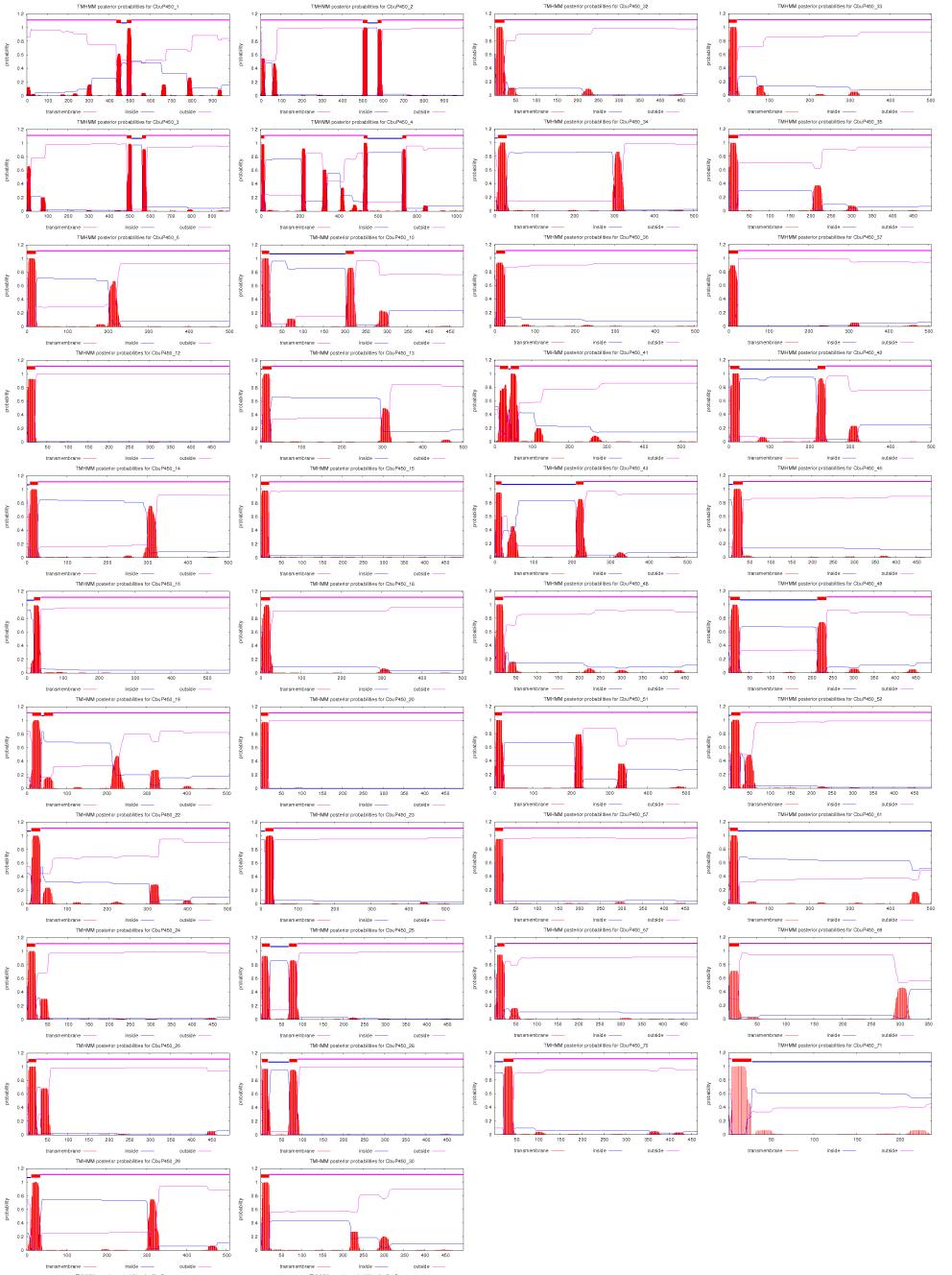


Supplementary Fig.1 Transmembrane domain analysis of CbuP450 protein family


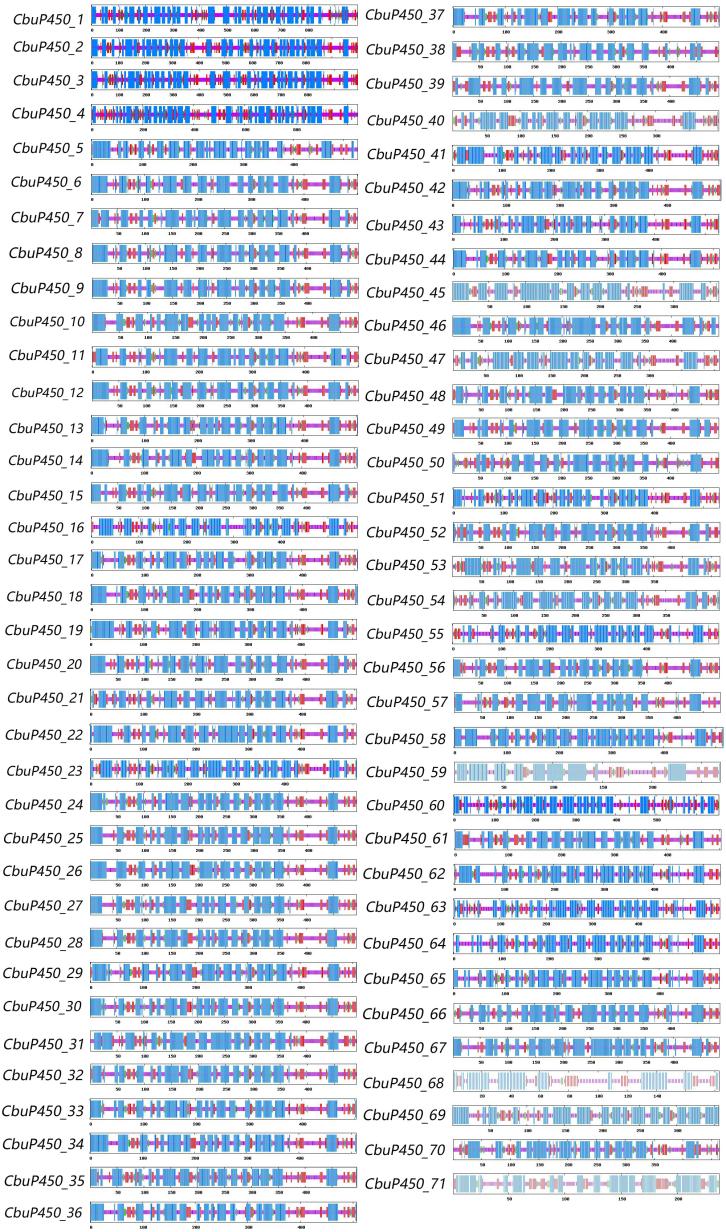


Supplementary Fig 2 Secondary structure analysis of CbuP450 protein family


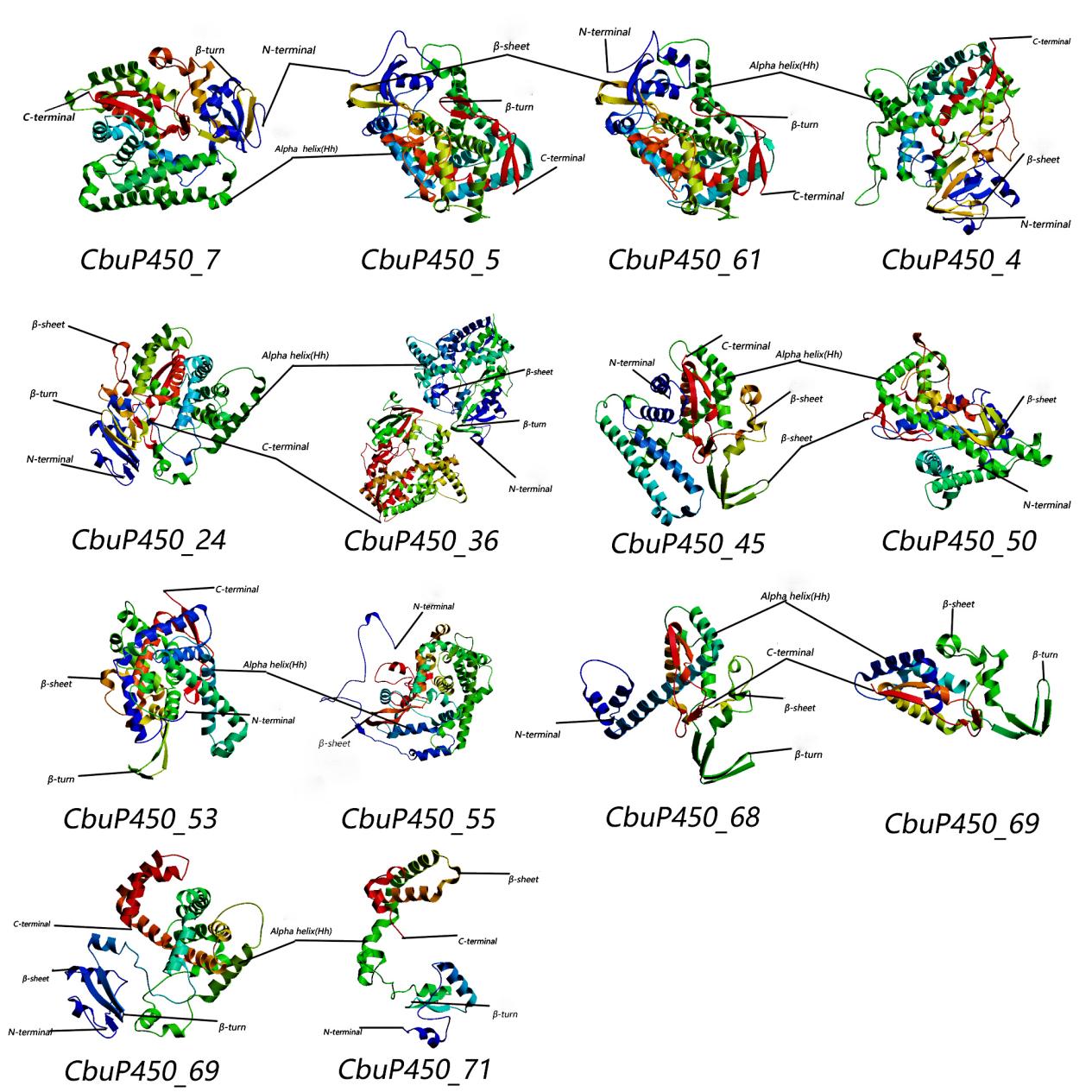


Supplementary Fig.3.Tertiary structure analysis of CbuP450 proteins


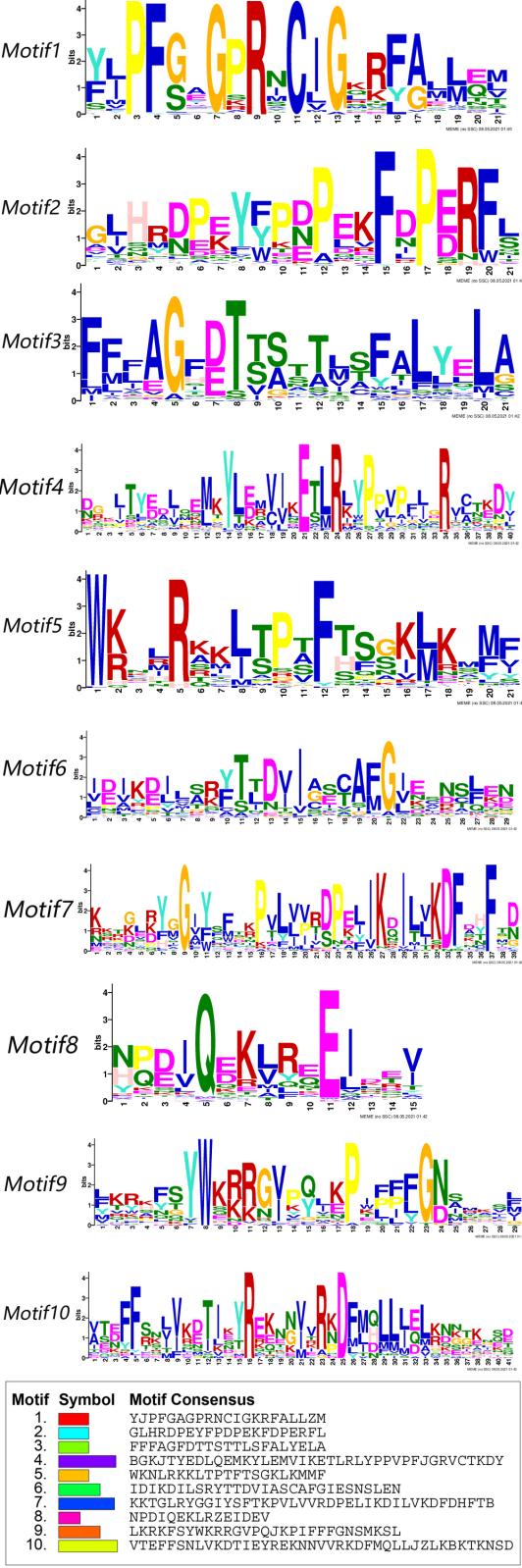

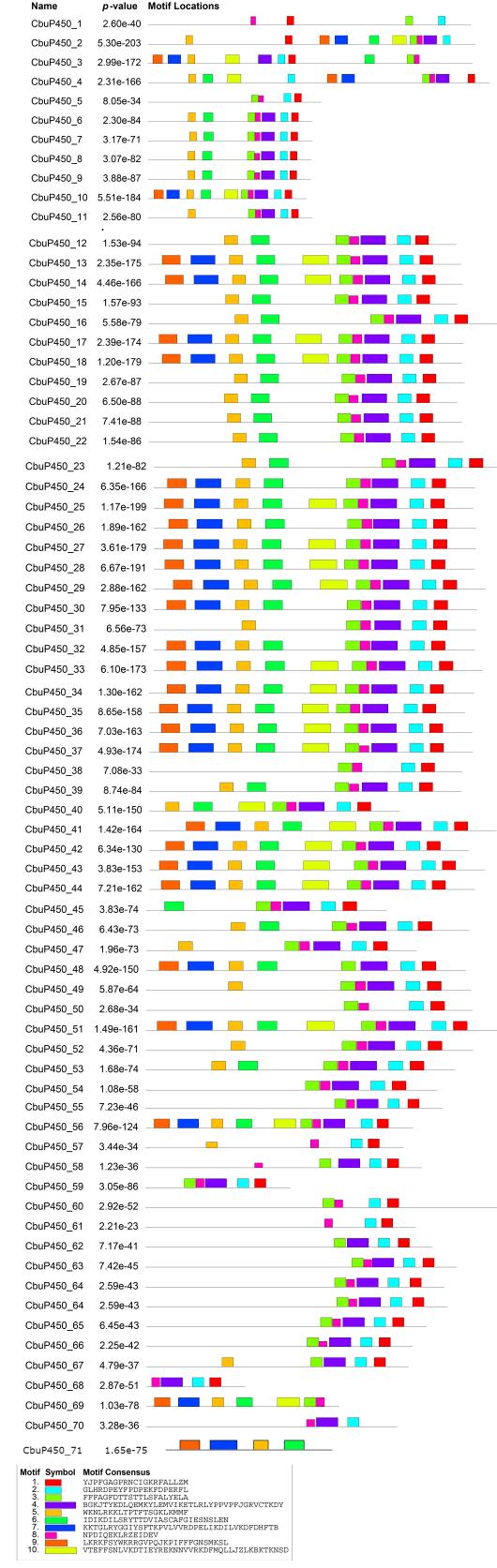


Supplementary Fig 4.Analysis of the conserved motifs of CbuP450 proteins


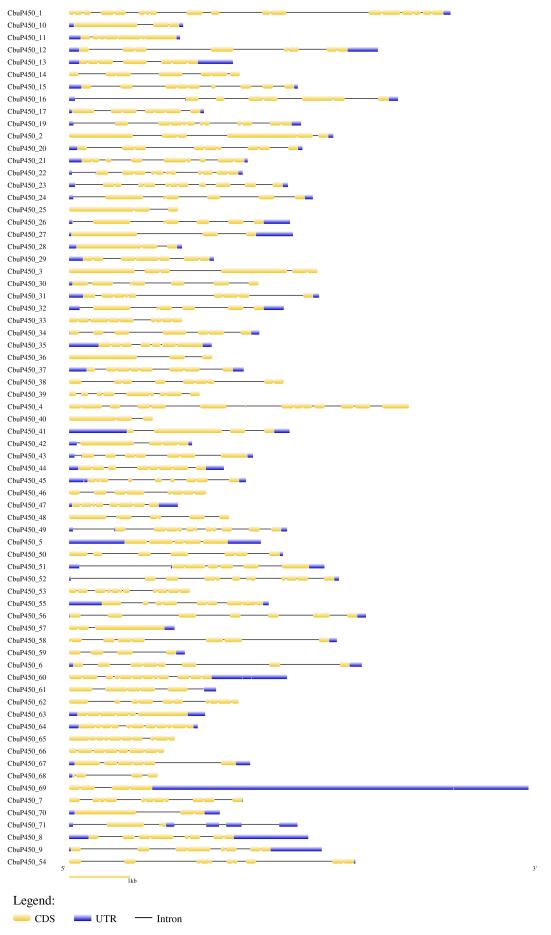


Supplementary Fig 5.Gene structure analysis of *P450* gene family in *C. buqueti*
