# Supplementary material for: Genome-wide identification, molecular evolution and gene expression of P450 gene family in Cyrtotrachelus buqueti
Source: BMC Genomics. 2024 May 8;25:453. doi: 10.1186/s12864-024-10372-5 (PMC11080265; doi:10.1186/s12864-024-10372-5)
Supplement: Supplementary file 1 — Supplementary Material 1. [file 12864_2024_10372_MOESM1_ESM.zip › Supplementary file/Supplementary table1-4.docx]

Supplementary Table 1 Protein characteristics of CbuP450 protein family

| Protein Name | Protein ID | Number amion  (aa） | Molecular weight(kD) | Theoreti cal pI | Instability index | Aliphatic index | Total number of negatively charged | Total positive residue | Grand average of hydropathicity | Subcellular localization |
| --- | --- | --- | --- | --- | --- | --- | --- | --- | --- | --- |
| CbuP450_1 | EVM0008092.1 | 984 | 113.26 | 8.84 | 42.66 | 97.45 | 107 | 123 | -0.129 | Plasma Membrane |
| CbuP450_2 | EVM0005211.1 | 997 | 115.56 | 8.71 | 38.79 | 90.8 | 116 | 128 | -0.186 | Plasma Membrane |
| CbuP450_3 | EVM0011422.1 | 988 | 114.16 | 8.8 | 32.08 | 90.92 | 115 | 126 | -0.149 | Plasma Membrane |
| CbuP450_4 | EVM0011494.1 | 1041 | 121.00 | 7.99 | 31.56 | 85.52 | 134 | 137 | -0.231 | Plasma Membrane |
| CbuP450_5 | EVM0012130.1 | 528 | 60.68 | 7.06 | 42.83 | 98.35 | 64 | 63 | -0.133 | Plasma Membrane, Nuclear, Mitochondrial |
| CbuP450_6 | EVM0009796.1 | 501 | 57.83 | 8.5 | 30.33 | 88.12 | 57 | 62 | -0.172 | Plasma Membrane |
| CbuP450_7 | EVM0008610.1 | 500 | 58.30 | 7.57 | 46.71 | 86.92 | 63 | 64 | -0.298 | Plasma Membrane, Cytoplasmic |
| CbuP450_8 | EVM0008387.1 | 497 | 57.16 | 8.86 | 32.02 | 101.99 | 61 | 68 | -0.176 | Plasma Membrane |
| CbuP450_9 | EVM0004315.2 | 496 | 56.96 | 8.1 | 31 | 99.84 | 61 | 63 | -0.183 | Plasma Membrane |
| CbuP450_10 | EVM0011870.1 | 483 | 55.62 | 8.61 | 36.56 | 85.71 | 56 | 61 | -0.182 | Plasma Membrane |
| CbuP450_11 | EVM0007331.1 | 501 | 58.17 | 8.94 | 42.47 | 95.33 | 57 | 64 | -0.205 | Plasma Membrane, Mitochondrial,  Nuclear |
| CbuP450_12 | EVM0009367.1 | 495 | 57.19 | 8.66 | 33.61 | 99.05 | 62 | 69 | -0.167 | Plasma Membrane, Nuclear |
| CbuP450_13 | EVM0010697.1 | 502 | 58.52 | 6.85 | 37.16 | 91.39 | 65 | 64 | -0.279 | Plasma Membrane, Cytoplasmic, Nuclear |
| CbuP450_14 | EVM0008233.1 | 505 | 59.33 | 8.51 | 42.57 | 95.35 | 65 | 69 | -0.289 | Plasma Membrane |
| CbuP450_15 | EVM0004218.1 | 496 | 57.28 | 8.43 | 30.56 | 103.57 | 61 | 65 | -0.092 | Plasma Membrane |
| CbuP450_16 | EVM0002741.1 | 563 | 64.24 | 8.72 | 37.44 | 88.01 | 72 | 78 | -0.322 | Nuclear, Cytoplasmic, Mitochondrial |
| CbuP450_17 | EVM0005245.1 | 506 | 58.37 | 9 | 38.39 | 81.86 | 57 | 67 | -0.242 | Cytoplasmic |
| CbuP450_18 | EVM0006182.1 | 504 | 58.19 | 8.95 | 38.34 | 85.83 | 56 | 64 | -0.218 | Plasma Membrane |
| CbuP450_19 | EVM0006382.1 | 508 | 59.31 | 9.33 | 44.09 | 98.78 | 47 | 66 | -0.066 | Plasma Membrane |
| CbuP450_20 | EVM0011595.1 | 496 | 57.39 | 8.87 | 31.64 | 96.21 | 53 | 60 | -0.183 | Plasma Membrane |
| CbuP450_21 | EVM0004116.1 | 504 | 58.11 | 9.3 | 46.88 | 98.45 | 51 | 66 | -0.161 | Plasma Membrane |
| CbuP450_22 | EVM0008642.1 | 506 | 59.13 | 9.25 | 42 | 99.94 | 48 | 65 | -0.047 | Plasma Membrane |
| CbuP450_23 | EVM0010968.1 | 550 | 63.28 | 7.65 | 45.06 | 90.95 | 72 | 73 | -0.282 | Cytoplasmic |
| CbuP450_24 | EVM0009842.2 | 492 | 57.09 | 8.68 | 42.71 | 91.32 | 53 | 60 | -0.057 | Plasma Membrane |
| CbuP450_25 | EVM0003714.1 | 490 | 56.67 | 8.65 | 38.25 | 94.27 | 55 | 61 | -0.084 | Plasma Membrane |
| CbuP450_26 | EVM0002245.1 | 494 | 57.57 | 8.84 | 38.49 | 92.53 | 52 | 61 | -0.095 | Plasma Membrane |
| CbuP450_27 | EVM0011735.1 | 493 | 57.07 | 8.85 | 37.61 | 88.38 | 55 | 62 | -0.174 | Plasma Membrane |
| CbuP450_28 | EVM0004729.1 | 491 | 57.14 | 8.53 | 35.8 | 91.89 | 57 | 62 | -0.133 | Plasma Membrane |
| CbuP450_29 | EVM0002036.1 | 508 | 59.05 | 8.62 | 38.74 | 87.99 | 63 | 69 | -0.17 | Plasma Membrane |
| CbuP450_30 | EVM0003907.1 | 495 | 57.26 | 8.95 | 35.05 | 99.25 | 53 | 64 | -0.057 | Plasma Membrane |
| CbuP450_31 | EVM0006264.1 | 493 | 56.56 | 9.06 | 35.37 | 101.22 | 47 | 59 | -0.011 | Plasma Membrane |
| CbuP450_32 | EVM0007774.2 | 491 | 56.81 | 8.74 | 40.74 | 96.86 | 50 | 58 | 0.001 | Plasma Membrane |
| CbuP450_33 | EVM0008111.1 | 503 | 58.37 | 8.99 | 38.3 | 82.72 | 62 | 74 | -0.333 | Cytoplasmic, Mitochondrial |
| CbuP450_34 | EVM0004655.1 | 510 | 59.22 | 8.83 | 38.08 | 88.45 | 57 | 63 | -0.189 | Plasma Membrane |
| CbuP450_35 | EVM0000515.1 | 496 | 58.07 | 9.15 | 35.96 | 92.74 | 55 | 65 | -0.151 | Plasma Membrane |
| CbuP450_36 | EVM0012061.1 | 507 | 59.04 | 9.08 | 34.97 | 88.82 | 54 | 66 | -0.221 | Plasma Membrane |
| CbuP450_37 | EVM0006938.1 | 508 | 58.26 | 8.48 | 42.28 | 90.41 | 63 | 68 | -0.119 | Plasma Membrane, Cytoplasmic |
| CbuP450_38 | EVM0002249.1 | 491 | 56.70 | 8.7 | 46.34 | 86.58 | 55 | 61 | -0.196 | Plasma Membrane |
| CbuP450_39 | EVM0007088.1 | 490 | 56.13 | 8.89 | 45.01 | 94.1 | 52 | 61 | -0.146 | Plasma Membrane, Mitochondrial |
| CbuP450_40 | EVM0002270.1 | 393 | 45.35 | 6.29 | 38.82 | 92.98 | 50 | 47 | -0.136 | Plasma Membrane |
| CbuP450_41 | EVM0001780.1 | 545 | 63.27 | 9.31 | 35.38 | 90.51 | 50 | 69 | 90.51 | Plasma Membrane |
| CbuP450_42 | EVM0011419.1 | 502 | 58.73 | 8.61 | 35.56 | 84.62 | 58 | 64 | -0.204 | Plasma Membrane |
| CbuP450_43 | EVM0006586.1 | 527 | 61.42 | 8.43 | 40.68 | 89.85 | 65 | 69 | -0.184 | Plasma Membrane |
| CbuP450_44 | EVM0011786.1 | 511 | 59.25 | 9.03 | 36.29 | 84.44 | 59 | 70 | -0.287 | Cytoplasmic |
| CbuP450_45 | EVM0002644.1 | 362 | 42.31 | 5.93 | 41.5 | 91.8 | 56 | 50 | -0.254 | Cytoplasmic |
| CbuP450_46 | EVM0001738.1 | 486 | 56.89 | 9.3 | 34.27 | 102.7 | 52 | 70 | -0.133 | Plasma Membrane, Nuclear |
| CbuP450_47 | EVM0002982.1 | 407 | 47.19 | 8.97 | 44.14 | 96.02 | 46 | 54 | -0.19 | Plasma Membrane, Nuclear |
| CbuP450_48 | EVM0009749.1 | 481 | 56.11 | 9.16 | 36.03 | 87.34 | 45 | 59 | -0.079 | Plasma Membrane |
| CbuP450_49 | EVM0002308.1 | 489 | 56.42 | 8.54 | 30.93 | 100.82 | 53 | 58 | -0.042 | Plasma Membrane |
| CbuP450_50 | EVM0005999.1 | 491 | 56.73 | 6.34 | 37.29 | 92.53 | 64 | 60 | -0.301 | Cytoplasmic |
| CbuP450_51 | EVM0011797.3 | 531 | 62.01 | 7.64 | 29.76 | 83.73 | 67 | 68 | -0.304 | Plasma Membrane |
| CbuP450_52 | EVM0010654.1 | 492 | 56.85 | 8.42 | 46.58 | 96.08 | 57 | 61 | -0.126 | Plasma Membrane |
| CbuP450_53 | EVM0008368.1 | 465 | 53.76 | 8.57 | 38.04 | 98.3 | 49 | 54 | -0.073 | Plasma Membrane |
| CbuP450_54 | EVM0010161.1 | 438 | 52.15 | 8.32 | 54.9 | 89.45 | 52 | 55 | -0.31 | Plasma Membrane |
| CbuP450_55 | EVM0002547.1 | 552 | 63.48 | 8.74 | 38.78 | 85.13 | 65 | 71 | -0.372 | Mitochondrial |
| CbuP450_56 | EVM0005337.1 | 498 | 58.12 | 8.73 | 41.51 | 84.88 | 56 | 63 | -0.297 | Plasma Membrane |
| CbuP450_57 | EVM0005525.1 | 480 | 54.81 | 7.14 | 39.61 | 93.19 | 48 | 48 | -0.053 | Plasma Membrane |
| CbuP450_58 | EVM0000696.1 | 514 | 58.26 | 9.06 | 41.6 | 99.03 | 53 | 63 | -0.068 | Plasma Membrane |
| CbuP450_59 | EVM0010729.1 | 269 | 31.45 | 7 | 37.95 | 97.1 | 31 | 31 | -0.208 | Nuclear |
| CbuP450_60 | EVM0000618.1 | 655 | 75.08 | 7.2 | 49.64 | 92.43 | 80 | 80 | -0.247 | Cytoplasmic, Nuclear |
| CbuP450_61 | EVM0002062.1 | 503 | 58.23 | 8.44 | 46.06 | 87.34 | 61 | 65 | -0.252 | Cytoplasmic |
| CbuP450_62 | EVM0012401.1 | 534 | 61.38 | 9.28 | 35.57 | 88.16 | 57 | 71 | -0.29 | Mitochondria |
| CbuP450_63 | EVM0012425.2 | 579 | 65.90 | 9.03 | 41.23 | 91.45 | 69 | 81 | -0.222 | Mitochondrial |
| CbuP450_64 | EVM0004101.1 | 556 | 64.31 | 8.9 | 49.16 | 93.24 | 63 | 72 | -0.294 | Cytoplasmic, Mitochondrial |
| CbuP450_65 | EVM0006390.1 | 518 | 59.92 | 8.54 | 47.71 | 94.05 | 68 | 73 | -0.254 | Cytoplasmic |
| CbuP450_66 | EVM0004649.1 | 492 | 56.54 | 9.26 | 42.65 | 102.01 | 52 | 66 | -0.231 | Mitochondrial |
| CbuP450_67 | EVM0004145.3 | 484 | 55.22 | 7.92 | 36.29 | 91.88 | 61 | 63 | -0.167 | Plasma Membrane, Mitochondrial |
| CbuP450_68 | EVM0006134.1 | 183 | 21.41 | 8.28 | 49.15 | 90.6 | 24 | 26 | -0.297 | Cytoplasmic, Nuclear |
| CbuP450_69 | EVM0004850.1 | 356 | 41.21 | 8.64 | 35.92 | 91.15 | 38 | 42 | -0.158 | Plasma Membrane |
| CbuP450_70 | EVM0005284.1 | 464 | 54.00 | 6.37 | 34.05 | 104.57 | 59 | 57 | -0.09 | Plasma Membrane |
| CbuP450_71 | EVM0001891.1 | 237 | 27.53 | 9.41 | 36.69 | 83.08 | 18 | 29 | 0.024 | Plasma Membrane |

Supplementary Table 2 The hydrophobicity analysis of amino acids

| Protein name | Grand average of hydropathicity | Maximum hydrophobicity | | Maximum hydrophilicity | |
| --- | --- | --- | --- | --- | --- |
|  |  | Position | Value | Position | Value |
| CbuP450_1 | -0.129 | 6 | 3.822 | 891 | -2.944 |
| CbuP450_2 | -0.186 | 515 | 3.244 | 411, 412 | -3.256 |
| CbuP450_3 | -0.149 | 7, 501 | 3.911 | 23, 517 | -3.344 |
| CbuP450_4 | -0.231 | 9 | 3.456 | 447 | -2.744 |
| CbuP450_5 | -0.133 | 28 | 3.722 | 307 | -3.022 |
| CbuP450_6 | -0.172 | 15 | 3.644 | 199 | -2.667 |
| CbuP450_7 | -0.298 | 12 | 3.544 | 264 | -2.689 |
| CbuP450_8 | -0.176 | 11 | 3.644 | 410 | -3.122 |
| CbuP450_9 | -0.183 | 11 | 3.222 | 410 | -3.122 |
| CbuP450_10 | -0.182 | 11, 12 | 3.722 | 410, 411 | -3.078 |
| CbuP450_11 | -0.205 | 7 | 3.422 | 236, 237 | -2.811 |
| CbuP450_12 | -0.167 | 13 | 3.689 | 260, 261, 267 | -2.956 |
| CbuP450_13 | -0.279 | 12 | 3.289 | 431 | -3.211 |
| CbuP450_14 | -0.289 | 17 | 3.567 | 433 | -3.344 |
| CbuP450_15 | -0.092 | 8 | 3.522 | 26 | -3.067 |
| CbuP450_16 | -0.322 | 28 | 2.722 | 295 | -2.567 |
| CbuP450_17 | -0.242 | 308 | 2.711 | 127 | -3.211 |
| CbuP450_18 | -0.218 | 12 | 3.622 | 286 | -3.078 |
| CbuP450_19 | -0.066 | 22 | 3.389 | 274 | -3.278 |
| CbuP450_20 | -0.183 | 6, 7, 8 | 3.233 | 269 | -3.133 |
| CbuP450_21 | -0.161 | 10 | 4.189 | 236 | -2.811 |
| CbuP450_22 | -0.047 | 20 | 3.4 | 272 | -3.233 |
| CbuP450_23 | -0.282 | 26 | 3 | 458 | -2.889 |
| CbuP450_24 | -0.057 | 14 | 2.989 | 282 | -2.656 |
| CbuP450_25 | -0.084 | 8 | 3.356 | 413 | -2.6 |
| CbuP450_26 | -0.095 | 18 | 2.933 | 284 | -3 |
| CbuP450_27 | -0.174 | 9 | 3.256 | 198 | -2.911 |
| CbuP450_28 | -0.133 | 9 | 2.778 | 24 | -2.411 |
| CbuP450_29 | -0.17 | 24 | 3.722 | 125 | -3.133 |
| CbuP450_30 | -0.057 | 13 | 3.278 | 417, 418 | -2.856 |
| CbuP450_31 | -0.011 | 91 | 3.3 | 262 | -3.3 |
| CbuP450_32 | 0.001 | 15 | 2.933 | 281 | -2.656 |
| CbuP450_33 | -0.333 | 17 | 3.6 | 431 | -2.9 |
| CbuP450_34 | -0.189 | 310 | 3.022 | 292 | -2.844 |
| CbuP450_35 | -0.151 | 9 | 4.044 | 23, 425 | -2.589 |
| CbuP450_36 | -0.221 | 8 | 3.744 | 218 | -2.7 |
| CbuP450_37 | -0.119 | 6 | 3.9 | 261 | -2.889 |
| CbuP450_38 | -0.196 | 8, 9, 10 | 3.722 | 193 | -2.856 |
| CbuP450_39 | -0.146 | 309 | 2.522 | 418 | -2.644 |
| CbuP450_40 | -0.136 | 196 | 2.6 | 102 | -2.7 |
| CbuP450_41 | -0.140 | 47 | 3.744 | 257 | -2.733 |
| CbuP450_42 | -0.204 | 18 | 3.533 | 33 | -3.167 |
| CbuP450_43 | -0.184 | 10 | 2.844 | 280 | -3.356 |
| CbuP450_44 | -0.287 | 6 | 3.367 | 128 | -2.989 |
| CbuP450_45 | -0.254 | 184 | 2.289 | 119 | -3.278 |
| CbuP450_46 | -0.133 | 19 | 3.178 | 243 | -3.189 |
| CbuP450_47 | -0.19 | 5 | 2.967 | 176 | -3.344 |
| CbuP450_48 | -0.079 | 15 | 2.967 | 281 | -2.656 |
| CbuP450_49 | -0.042 | 12 | 3.022 | 398 | -2.878 |
| CbuP450_50 | -0.301 | 453 | 2.089 | 277, 278 | -3.456 |
| CbuP450_51 | -0.304 | 9 | 3.3 | 412 | -2.733 |
| CbuP450_52 | -0.126 | 22 | 2.933 | 131 | -3 |
| CbuP450_53 | -0.073 | 40 | 2.789 | 372 | -2.878 |
| CbuP450_54 | -0.31 | 254 | 2.933 | 345 | -2.878 |
| CbuP450_55 | -0.372 | 271 | 2.033 | 381 | -2.889 |
| CbuP450_56 | -0.297 | 11 | 3.356 | 124 | -3.1 |
| CbuP450_57 | -0.053 | 10 | 3.656 | 26 | -2.722 |
| CbuP450_58 | -0.068 | 220 | 2.767 | 364 | -3.478 |
| CbuP450_59 | -0.208 | 50 | 2.456 | 59 | -2.656 |
| CbuP450_60 | -0.247 | 330 | 2.567 | 237 | -2.778 |
| CbuP450_61 | -0.252 | 14 | 3.511 | 296 | -2.644 |
| CbuP450_62 | -0.29 | 270 | 2.033 | 376 | -2.833 |
| CbuP450_63 | -0.222 | 541 | 2.689 | 191 | -2.789 |
| CbuP450_64 | -0.294 | 352 | 2.856 | 377 | -3.044 |
| CbuP450_65 | -0.254 | 324 | 2.5 | 349 | -2.6 |
| CbuP450_66 | -0.231 | 455 | 2.978 | 95 | -2.667 |
| CbuP450_67 | -0.167 | 11 | 3.433 | 123 | -3.044 |
| CbuP450_68 | -0.297 | 5 | 1.956 | 92 | -2.422 |
| CbuP450_69 | -0.158 | 6, 7 | 3.544 | 123 | -2.867 |
| CbuP450_70 | -0.09 | 38 | 2.933 | 86 | -2.978 |
| CbuP450_71 | 0.024 | 186 | 2.911 | 200 | -2.322 |

Supplementary Table 3 Phosphorylation sites analysis of CbuP450 proteins

| Protein Name | Phosphorylation site | | | Serine(S) | | Threonine(T) | | Tyrosine(Y) | |
| --- | --- | --- | --- | --- | --- | --- | --- | --- | --- |
|  | S | T | Y | Max | position | Max | position | Max | position |
| CbuP450_1 | 57 | 27 | 24 | 0.998 | 256 | 0.889 | 481 | 0.98 | 827 |
| CbuP450_2 | 50 | 54 | 16 | 0.992 | 712 | 0.981 | 764 | 0.909 | 668 |
| CbuP450_3 | 45 | 58 | 16 | 0.986 | 344 | 0.994 | 771 | 0.953 | 573 |
| CbuP450_4 | 93 | 49 | 25 | 0.995 | 26 | 0.901 | 53 | 0.929 | 55 |
| CbuP450_5 | 37 | 15 | 3 | 0.997 | 34 | 0.89 | 72 | 0.735 | 366 |
| CbuP450_6 | 21 | 21 | 14 | 0.993 | 100 | 0.963 | 183 | 0.955 | 88 |
| CbuP450_7 | 31 | 20 | 17 | 0.998 | 263 | 0.898 | 367 | 0.95 | 481 |
| CbuP450_8 | 28 | 19 | 11 | 0.998 | 17 | 0.915 | 53 | 0.989 | 71 |
| CbuP450_9 | 35 | 22 | 9 | 0.998 | 17 | 0.915 | 63 | 0.989 | 71 |
| CbuP450_10 | 37 | 18 | 7 | 0.995 | 45 | 0.987 | 459 | 0.934 | 394 |
| CbuP450_11 | 45 | 19 | 5 | 0.99 | 31 | 0.987 | 75 | 0.871 | 414 |
| CbuP450_12 | 25 | 17 | 6 | 0.998 | 161 | 0.99 | 378 | 0.925 | 199 |
| CbuP450_13 | 31 | 15 | 10 | 0.997 | 26 | 0.768 | 99 | 0.988 | 65 |
| CbuP450_14 | 31 | 17 | 14 | 0.992 | 288 | 0.989 | 385 | 0.956 | 98 |
| CbuP450_15 | 32 | 23 | 8 | 0.997 | 167 | 0.985 | 255 | 0.881 | 94 |
| CbuP450_16 | 54 | 29 | 11 | 0.997 | 6 | 0.979 | 13 | 0.979 | 83 |
| CbuP450_17 | 19 | 33 | 10 | 0.998 | 428 | 0.986 | 128 | 0.951 | 415 |
| CbuP450_18 | 36 | 24 | 13 | 0.995 | 54 | 0.908 | 99 | 0.988 | 496 |
| CbuP450_19 | 18 | 18 | 9 | 0.92 | 110 | 0.887 | 224 | 0.872 | 70 |
| CbuP450_20 | 38 | 19 | 8 | 0.993 | 269 | 0.932 | 290 | 0.954 | 97 |
| CbuP450_21 | 36 | 19 | 11 | 0.995 | 245 | 0.939 | 486 | 0.989 | 352 |
| CbuP450_22 | 20 | 18 | 8 | 0.898 | 108 | 0.887 | 222 | 0.926 | 80 |
| CbuP450_23 | 40 | 27 | 10 | 0.995 | 63 | 0.902 | 202 | 0.944 | 459 |
| CbuP450_24 | 34 | 16 | 17 | 0.993 | 108 | 0.888 | 356 | 0.972 | 160 |
| CbuP450_25 | 23 | 31 | 6 | 0.991 | 205 | 0.94 | 257 | 0.909 | 161 |
| CbuP450_26 | 33 | 16 | 16 | 0.972 | 466 | 0.929 | 358 | 0.968 | 162 |
| CbuP450_27 | 17 | 21 | 12 | 0.985 | 278 | 0.902 | 358 | 0.962 | 376 |
| CbuP450_28 | 23 | 33 | 6 | 0.996 | 370 | 0.945 | 365 | 0.909 | 162 |
| CbuP450_29 | 26 | 16 | 13 | 0.998 | 432 | 0.963 | 76 | 0.883 | 141 |
| CbuP450_30 | 28 | 20 | 9 | 0.997 | 6 | 0.986 | 19 | 0.979 | 27 |
| CbuP450_31 | 32 | 15 | 2 | 0.997 | 235 | 0.964 | 375 | 0.853 | 446 |
| CbuP450_32 | 38 | 14 | 16 | 0.996 | 107 | 0.929 | 355 | 0.955 | 70 |
| CbuP450_33 | 28 | 34 | 11 | 0.998 | 426 | 0.969 | 477 | 0.878 | 413 |
| CbuP450_34 | 22 | 25 | 7 | 0.997 | 177 | 0.898 | 34 | 0.903 | 139 |
| CbuP450_35 | 31 | 23 | 11 | 0.997 | 22 | 0.926 | 90 | 0.903 | 54 |
| CbuP450_36 | 19 | 17 | 11 | 0.98 | 477 | 0.893 | 370 | 0.969 | 126 |
| CbuP450_37 | 28 | 19 | 11 | 0.976 | 163 | 0.885 | 371 | 0.975 | 389 |
| CbuP450_38 | 43 | 15 | 7 | 0.998 | 49 | 0.972 | 18 | 0.926 | 255 |
| CbuP450_39 | 29 | 14 | 6 | 0.996 | 232 | 0.981 | 463 | 0.971 | 258 |
| CbuP450_40 | 24 | 20 | 6 | 0.997 | 21 | 0.876 | 257 | 0.909 | 64 |
| CbuP450_41 | 20 | 19 | 10 | 0.98 | 516 | 0.959 | 469 | 0.969 | 165 |
| CbuP450_42 | 30 | 21 | 9 | 0.985 | 207 | 0.888 | 366 | 0.947 | 384 |
| CbuP450_43 | 30 | 24 | 14 | 0.993 | 41 | 0.943 | 32 | 0.933 | 60 |
| CbuP450_44 | 26 | 31 | 14 | 0.997 | 433 | 0.975 | 134 | 0.968 | 490 |
| CbuP450_45 | 21 | 14 | 5 | 0.997 | 336 | 0.907 | 42 | 0.703 | 334 |
| CbuP450_46 | 37 | 15 | 6 | 0.998 | 481 | 0.912 | 409 | 0.957 | 441 |
| CbuP450_47 | 34 | 12 | 2 | 0.997 | 331 | 0.964 | 289 | 0.912 | 360 |
| CbuP450_48 | 36 | 10 | 12 | 0.993 | 107 | 0.917 | 345 | 0.972 | 159 |
| CbuP450_49 | 24 | 21 | 6 | 0.995 | 21 | 0.99 | 4 | 0.927 | 399 |
| CbuP450_50 | 34 | 21 | 12 | 0.997 | 7 | 0.983 | 16 | 0.858 | 24 |
| CbuP450_51 | 48 | 23 | 10 | 0.997 | 26 | 0.849 | 45 | 0.929 | 60 |
| CbuP450_52 | 41 | 29 | 12 | 0.997 | 464 | 0.935 | 58 | 0.921 | 462 |
| CbuP450_53 | 34 | 18 | 7 | 0.992 | 11 | 0.971 | 22 | 0.942 | 73 |
| CbuP450_54 | 29 | 12 | 13 | 0.997 | 37 | 0.814 | 24 | 0.933 | 12 |
| CbuP450_55 | 49 | 21 | 9 | 0.998 | 429 | 0.981 | 475 | 0.851 | 47 |
| CbuP450_56 | 35 | 19 | 16 | 0.995 | 160 | 0.935 | 251 | 0.992 | 295 |
| CbuP450_57 | 32 | 18 | 9 | 0.978 | 20 | 0.959 | 21 | 0.876 | 135 |
| CbuP450_58 | 37 | 20 | 8 | 0.996 | 485 | 0.958 | 119 | 0.833 | 130 |
| CbuP450_59 | 11 | 9 | 10 | 0.904 | 133 | 0.859 | 223 | 0.945 | 125 |
| CbuP450_61 | 43 | 14 | 13 | 0.996 | 356 | 0.803 | 136 | 0.971 | 268 |
| CbuP450_62 | 44 | 17 | 12 | 0.998 | 411 | 0.981 | 457 | 0.874 | 441 |
| CbuP450_63 | 31 | 23 | 2 | 0.978 | 483 | 0.992 | 347 | 0.976 | 474 |
| CbuP450_64 | 41 | 30 | 6 | 0.991 | 395 | 0.924 | 57 | 0.858 | 105 |
| CbuP450_65 | 45 | 20 | 8 | 0.993 | 447 | 0.962 | 293 | 0.992 | 429 |
| CbuP450_66 | 39 | 11 | 6 | 0.995 | 198 | 0.954 | 389 | 0.908 | 234 |
| CbuP450_67 | 47 | 27 | 6 | 0.998 | 25 | 0.893 | 105 | 0.949 | 29 |
| CbuP450_68 | 11 | 6 | 8 | 0.997 | 157 | 0.633 | 168 | 0.921 | 155 |
| CbuP450_69 | 16 | 14 | 10 | 0.986 | 21 | 0.991 | 74 | 0.888 | 64 |
| CbuP450_70 | 44 | 15 | 10 | 0.997 | 316 | 0.916 | 336 | 0.965 | 328 |
| CbuP450_71 | 27 | 10 | 6 | 0.981 | 157 | 0.914 | 65 | 0.982 | 163 |

Supplementary Table 4 Secondary structure analysis of CbuP450 protein family

| Protein name | α-helix | Extended chain | β-turn | Random coil |
| --- | --- | --- | --- | --- |
| CbuP450_1 | 438(44.51%) | 108(10.98%) | 58(5.89%) | 380(38.62%) |
| CbuP450_2 | 470(47.14%) | 131(13.14%) | 40(4.01%) | 356(35.71%) |
| CbuP450_3 | 458(46.36%) | 132(13.36%) | 50(50.06%) | 348(35.22%) |
| CbuP450_4 | 453(43.52%) | 156(14.99%) | 65(6.24%) | 367(35.25%) |
| CbuP450_5 | 256(48.48%) | 58(10.98%) | 21(3.98%) | 193(36.55%) |
| CbuP450_6 | 241(48.10%) | 60(11.98%) | 17(3.39%) | 183(36.53%) |
| CbuP450_7 | 243(48.60%) | 55(11.00%) | 19(3.80%) | 183(36.60%) |
| CbuP450_8 | 237(47.69%) | 61(12.27%) | 19(3.82%) | 180(36.22%) |
| CbuP450_9 | 243(48.99%) | 59(11.90%) | 20(4.03%) | 174(35.08%) |
| CbuP450_10 | 225(46.58%) | 68(14.08%) | 27(5.59%) | 163(33.75%) |
| CbuP450_11 | 231(46.11%) | 69.13.77%) | 17( 3.39%) | 184(36.73%) |
| CbuP450_12 | 243(49.09%) | 57(11.52%) | 25(5.05%) | 170(34.34%) |
| CbuP450_13 | 235(46.81%) | 68(13.55%) | 22(4.38%) | 177(35.26%) |
| CbuP450_14 | 241(47.72%) | 69(13.66%) | 17(3.37%) | 178(35.25%) |
| CbuP450_15 | 246(49.60%) | 59(11.90%) | 19(3.83%) | 172(34.68%) |
| CbuP450_16 | 256(45.47%) | 62(11.01%) | 22(3.91%) | 223(39.61%) |
| CbuP450_17 | 228(45.06%) | 67(13.24%) | 23(4.55%) | 188(37.15%) |
| CbuP450_18 | 240(47.62%) | 72(14.29%) | 18(3.57%) | 174(34.52%) |
| CbuP450_19 | 260(51.18%) | 65(12.80%) | 17(3.35%) | 166(32.68%) |
| CbuP450_20 | 241(48.59%) | 64(12.90%) | 19(3.83%) | 172( 34.68%) |
| CbuP450_21 | 230(45.63%) | 70(13.89%) | 17(3.37%） | 187(37.10%） |
| CbuP450_22 | 264(52.17%） | 63(12.45%） | 12(2.37%） | 167(33.00%） |
| CbuP450_23 | 280(50.91%） | 66(12.00%） | 31(5.64%） | 173(31.45%） |
| CbuP450_24 | 233(47.36%） | 60(12.20%） | 29(5.89%） | 170(34.55%） |
| CbuP450_25 | 232(47.35%） | 70(14.29%） | 15(3.06%） | 173(35.31%） |
| CbuP450_26 | 247(50.00%） | 61(12.35%） | 19(3.85%） | 167(33.81%） |
| CbuP450_27 | 220(44.62%） | 70(14.20%） | 20(4.06%) | 183(37.12%) |
| CbuP450_28 | 225(45.82%) | 69(14.05%) | 21(4.28%) | 176(35.85%） |
| CbuP450_29 | 240(47.24%) | 72(14.17%) | 22(4.33%) | 174(34.25%) |
| CbuP450_30 | 229(46.26%) | 69(13.94%) | 32(6.46%） | 165(33.33%) |
| CbuP450_31 | 254(51.52%) | 67(13.59%） | 21(4.26%) | 151(30.63%） |
| CbuP450_32 | 235(47.86%) | 63(12.83%） | 23(4.68%) | 170(34.62%) |
| CbuP450_33 | 234(46.51%) | 61(12.13%） | 27(5.37%) | 181(35.98%) |
| CbuP450_34 | 248(48.63%) | 67(13.14%) | 18(3.53%) | 177(34.71%) |
| CbuP450_35 | 234(47.18%) | 76(15.32%) | 23(4.64%) | 163(32.86%) |
| CbuP450_36 | 228(44.97%) | 68(13.41%） | 17(3.35%） | 194(38.26%) |
| CbuP450_37 | 235(46.26%) | 69(13.58%) | 24(4.72%) | 180(35.43%） |
| CbuP450_38 | 216(43.99%） | 62(12.63%) | 25(5.09%) | 188(38.29%) |
| CbuP450_39 | 223(45.51%) | 62(12.65%) | 19(3.88%) | 186(37.96%） |
| CbuP450_40 | 177(45.04%） | 60(15.27%） | 19(4.83%) | 137(34.86%） |
| CbuP450_41 | 264(48.44%） | 78(14.31%) | 24(4.40%) | 179(32.84%） |
| CbuP450_42 | 241(48.01%) | 68(13.55%) | 23(4.58%) | 170(33.86%） |
| CbuP450_43 | 227(43.07%) | 76(14.42%) | 26(4.93%) | 194(37.57%) |
| CbuP450_44 | 234(45.79%) | 70(13.70%) | 22(4.31%) | 185(36.20%） |
| CbuP450_45 | 194(53.59%） | 44(12.15%) | 17(4.70%) | 107(29.56%） |
| CbuP450_46 | 264(54.32%） | 55(11.32%) | 22(4.53%) | 145(29.84%） |
| CbuP450_47 | 203(49.88%） | 52(12.78%) | 17(4.18%) | 135(33.17%） |
| CbuP450_48 | 218(45.32%） | 75(15.59%) | 24(4.99%) | 164(34.10%) |
| CbuP450_49 | 237(48.47%) | 60(12.27%） | 14(2.86%） | 178(36.40%) |
| CbuP450_50 | 210(42.77%） | 63(12.83%） | 32(6.52%） | 186(37.88%） |
| CbuP450_51 | 235(44.26%) | 79(14.88%) | 25(4.71%) | 192(36.16%) |
| CbuP450_52 | 234(47.56%) | 72(14.63%) | 16(3.25%） | 170(34.55%） |
| CbuP450_53 | 224(48.17%) | 75(16.13%) | 25(5.38%) | 141(30.32%） |
| CbuP450_54 | 218(47.95%） | 57(13.01%) | 24(5.48%) | 147(33.56%） |
| CbuP450_55 | 243(44.02%) | 61(11.05%） | 24(4.35%) | 224(40.58%) |
| CbuP450_56 | 216(43.78%） | 74(14.86%) | 23(4.62%) | 183(36.75%) |
| CbuP450_57 | 211(43.96%） | 55(11.46%) | 19(3.96%） | 195(40.62%) |
| CbuP450_58 | 286(55.64%） | 42(8.17%) | 19(3.70%) | 167(32.49%） |
| CbuP450_59 | 119(44.24%） | 36(13.38%) | 12(4.46%） | 102(37.92%） |
| CbuP450_61 | 299(45.65%) | 71(10.84%) | 26(3.97%) | 259(39.54%) |
| CbuP450_62 | 226(44.93%) | 61(12.13%） | 21(4.17%) | 195(38.77%) |
| CbuP450_63 | 257(48.13%) | 48(8.99%) | 27(5.06%) | 202(37.83%) |
| CbuP450_64 | 268(46.29%） | 60(10.36%) | 28(4.84%) | 223(38.51%) |
| CbuP450_65 | 252(45.32%) | 52(9.35%) | 20(3.60%) | 232(41.73%) |
| CbuP450_66 | 255(49.23%) | 48(9.27%) | 23(4.44%) | 192(37.07%) |
| CbuP450_67 | 239(48.58%) | 52(10.57%) | 22(4.47%) | 179(36.38%） |
| CbuP450_68 | 252(52.07%) | 48(9.92%) | 25(5.17%) | 159(32.85%） |
| CbuP450_70 | 209(58.71%） | 31(8.71%) | 18(5.06%) | 98(27.53%) |
| CbuP450_71 | 212(45.69%） | 60(12.93%） | 26(5.60%) | 166(35.78%) |
